# Application of the electronic scoliometer in school-based scoliosis screening among elementary school students and analysis of factors associated with suspected scoliosis

**DOI:** 10.3389/fpubh.2026.1926931

**Published:** 2026-09-21

**Authors:** Yujie Guan, Baocheng Lin, Gege Wang, Qi Qin, Bin Zhao, Yuhuang Lin

**Affiliations:** 1 The Second Clinical College, Heilongjiang University of Chinese Medicine, Harbin, China; 2 Department of Soft Tissue Injury, Shenzhen Traditional Chinese Medicine Hospital, Shenzhen, China; 3 Department of Musculoskeletal Pain, The Second Affiliated Hospital of Heilongjiang University of Chinese Medicine, Harbin, China; 4 Department of Rehabilitation, Sanya Hospital of Traditional Chinese Medicine (Hainan Hospital, Guangzhou University of Chinese Medicine), Sanya, China

**Keywords:** cross-sectional study, electronic scoliometer, elementary school children, risk factors, school-based screening, scoliosis

## Abstract

**Background:**

Scoliosis imposes a growing health burden on school-age children in China, yet data specific to elementary school students aged 6–12 years remain scarce. This study aimed to compare the positive screening rate and screening efficiency of the electronic scoliometer (E-scoliometer) with those of the manual scoliometer (M-scoliometer), and to identify modifiable risk factors for suspected scoliosis in this younger age group.

**Methods:**

A school-based cross-sectional study was conducted among 11,587 students in grades 1–5 from 10 public elementary schools in Harbin, China (September 2024–May 2025), using multistage stratified cluster sampling. Classes were randomly allocated to the M-scoliometer group (*n* = 5,697 pupils) or the E-scoliometer group (*n* = 5,890). A stratified subsample of 3,600 students underwent posture assessment and parent-reported questionnaire survey. Associations were examined within two domains by binary logistic regression and then in a single joint generalized estimating equation (GEE) model providing mutual adjustment across domains and accounting for clustering of pupils within classes.

**Results:**

The overall positive screening rate was 1.61%, increasing significantly from Grade 1 (0.26%) to Grade 5 (2.89%; *p* < 0.001). The two devices yielded comparable positive screening rates (M-scoliometer 1.67% vs. E-scoliometer 1.56%; difference 0.11 percentage points, 95% CI − 0.36 to 0.57; *p* = 0.706), while the E-scoliometer reduced mean screening time by 54.2% (16.04 s vs. 35.00 s; *p* < 0.001). In the joint GEE model (59 events, 9 parameters, 6.56 events per variable, optimism-corrected AUC 0.910), flat back (OR = 7.36), picky eating (OR = 4.19), daily physical exercise < 1 h/day (OR = 3.86), mild wasting (OR = 3.80), shoulder height asymmetry (OR = 3.06) and higher grade (OR = 1.43 per grade) were associated with higher odds of suspected scoliosis, whereas regular adjustment of chair and desk height (OR = 0.18) and regular rotation of classroom seating (OR = 0.19) were associated with lower odds. The association with thumb-inward grip pen-holding posture did not persist under mutual adjustment (OR = 1.55, 95% CI 0.77–3.11) and is reported as hypothesis-generating.

**Conclusion:**

The E-scoliometer yielded a positive screening rate comparable to that of the M-scoliometer with markedly superior efficiency, supporting its adoption in large-scale school-based programs. The factors identified were largely modifiable, suggesting that promoting daily physical activity, improving nutritional behaviors, and standardizing classroom furniture management and seating rotation merit consideration in school-based scoliosis prevention strategies for elementary school children.

## 1 Introduction

Scoliosis is a common musculoskeletal disorder characterized by three-dimensional deformity (coronal, sagittal, and axial planes), with a diagnostic criterion of a coronal plane Cobb angle of ≥10° on an anteroposterior full-spine X-ray taken in the standing position (1, 2). Among these, idiopathic scoliosis (IS) has an unknown cause and accounts for approximately 80–85% of all cases (3). Adolescent idiopathic scoliosis (AIS) is the most common type of IS, predominantly affecting adolescents aged 10–18 years, with a higher prevalence in females than in males (4, 5). Recent large-scale epidemiological studies have highlighted the growing burden of scoliosis among children and adolescents (6–8). A global systematic review and meta-analysis of 239 studies reported a pooled prevalence of approximately 1.65%, with a continuous upward trend since 2020 (6). In China, scoliosis likewise imposes a considerable burden on school-age children: a population-wide analysis of over 2.2 million students reported a positive rate of 3.97% on school-based scoliosis screening (SSS), a confirmed prevalence of approximately 1.20%, and a prevalence in girls 1.57 times that in boys (7). Notably, the prevalence of scoliosis among Chinese primary and secondary school students increased significantly between 2016 and 2024 compared to the period from 2000 to 2015, and the disease burden continues to grow (8).

Failure to detect scoliosis early and intervene promptly can lead to a variety of adverse consequences. In addition to cosmetic deformities, progressive scoliosis can cause back pain, impaired lung function, mobility limitations, and psychological distress. Studies have confirmed that the prevalence of anxiety and depression symptoms is significantly higher among patients with AIS than in healthy controls (9), and those whose curvature progresses into adulthood also face long-term risks such as chronic pain, osteoporosis, and reduced cardiopulmonary function (10). Early detection and control of scoliosis at the mild stage are key to improving individual health outcomes and reducing the subsequent medical burden (9, 11).

The Adams forward bend test combined with a scoliometer to measure the angle of trunk rotation (ATR) is currently the most widely used method for SSS (12–15). However, manual screening scales poorly: inter-examiner variability in ATR measurements can reach 4°–5° (and is even greater in the lumbar segment), and the method depends heavily on the availability of trained personnel (16). Digital-sensor-based electronic scoliometers (E-scoliometers) have been introduced to address these limitations, and existing studies suggest that the E-scoliometer can match or even surpass the M-scoliometer with respect to the speed, reliability, and measurement accuracy of ATR (17–19). However, this evidence derives largely from small, controlled validation studies (17); real-world application in large-scale school screening has emerged only recently (18, 19), including a large-scale comparison conducted predominantly in junior-high students (19), whereas the device’s performance and feasibility in younger elementary-school children (aged 6–12) — who are typically less cooperative and have smaller, more pliable trunks — remain insufficiently established. Clarifying whether the E-scoliometer is genuinely applicable to large-scale, real-world elementary screening is therefore of direct public-health relevance, as such programs must be fast, reliable, and feasible with limited personnel while detecting scoliosis at an earlier, more conservatively treatable stage.

Analysis of risk factors for scoliosis is a crucial foundation for developing targeted prevention and control strategies. Current evidence suggests that its occurrence is associated with various factors, including individual characteristics, poor posture, and lifestyle habits. Regarding posture, poor sitting posture (OR = 3.48) has been identified as one of the strongest independent risk factors (6). Adolescents exhibiting a rib hump in the thoracic spine have approximately 10 times the risk of developing scoliosis compared to those with normal posture (20), suggesting a strong association between postural abnormalities and scoliosis. Regarding lifestyle habits, prolonged sitting, excessive screen time, insufficient outdoor activity, and low Body Mass Index (BMI) have all been shown to be associated with higher prevalence rates (6, 21), while factors such as nutritional status and school seating environments have also garnered attention in recent studies (22). Nevertheless, existing studies have primarily focused on middle school-aged and older populations (≥12 years), with limited analyses of risk factors in younger elementary school-aged children (6–12 years).

Therefore, in collaboration with the local education bureau, we conducted SSS in grades 1–5 (aged 6–12) at 10 public schools in Harbin, Heilongjiang Province. This study had two integrated aims: first, to compare the positive screening rate, screening efficiency and real-world applicability of the E-scoliometer with those of the M-scoliometer in this elementary-age population; and second, to identify personal, postural, and lifestyle risk factors for suspected scoliosis in the same cohort. By addressing both questions within one large-scale elementary screening program, this study connects how to screen efficiently with whom to prioritize, providing integrated, actionable evidence for designing scalable school-based scoliosis screening and prevention strategies for elementary school children.

## 2 Methods

### 2.1 Study design and study population

This study was a school-based cross-sectional study conducted in Harbin, Heilongjiang Province, from September 2024 to May 2025, with the support of the local education bureau. A multistage, stratified, cluster sampling method was employed. First, five districts (four urban and one suburban) were stratified and selected from the nine administrative districts of Harbin. Subsequently, two public elementary schools were randomly selected from each of the selected districts, resulting in a total of 10 schools. Within each participating school, classes in each grade were randomly divided into equal groups and assigned to either the M-scoliometer group (*n* = 5,697) or the E-scoliometer group (*n* = 5,890). A total of 11,587 elementary school students in grades 1 through 5 were included and screened. To analyze the association between poor posture, daily habits, and suspected scoliosis, a stratified cluster sampling method by grade was further applied within the M-scoliometer group. Using classes as the sampling units, two classes were randomly selected from each grade in every school, resulting in a total of 3,600 students who underwent simultaneous posture assessment and questionnaire surveys. The overall study design and participant flow are illustrated in Supplementary Figure S1.

Inclusion criteria: (1) Enrolled students in grades 1–5 at sampled elementary schools in Harbin; (2) Aged 6–12 years; (3) Legal guardians voluntarily signed a written informed consent form. Exclusion criteria: (1) Individuals unable to complete the standard Adams forward bend test due to physical disabilities or other reasons; (2) Students absent on the day of screening.

### 2.2 Ethical approval

This study protocol was reviewed and approved in May 2024 by the Ethics Review Committee of the Second Affiliated Hospital of Heilongjiang University of Chinese Medicine (Approval no. 2025 K33), before the pilot study and the commencement of formal screening. All procedures complied with the ethical principles of the 2013 revised version of the Declaration of Helsinki. Before any study procedure was performed, including those of the pilot study, the participants’ legal guardians voluntarily signed written informed consent forms after fully understanding the study’s objectives and methods. Participants could withdraw unconditionally at any stage. All personal information was strictly confidential throughout the data collection and analysis process and was de-identified prior to statistical analysis.

### 2.3 Screening protocol

#### 2.3.1 Pre-screening preparation and training

All medical personnel involved in the screening (physicians, therapists, and graduate students) were required to complete standardized training covering the operational guidelines outlined in the “Screening of Abnormal Spinal Curvature of Children and Adolescents” (GB/T 16133–2014) (23), issued by the National Health Commission of the People’s Republic of China. Training consisted of two phases: theoretical instruction and practical assessment. Personnel were permitted to begin work only after passing a standardized assessment. All measuring instruments were calibrated prior to the start of screening at each school.

Prior to formal screening, the research team conducted a pilot study in July 2024 in two randomly selected classes at a non-participating school (n = 86), covered by the same ethical approval and preceded by written guardian consent, to assess the measurement reliability of each device and the agreement between them. Reliability was evaluated separately for the two devices: intra-rater reliability (test–retest) was assessed by having a single trained examiner measure each participant twice under identical conditions, and inter-rater reliability by having two examiners measure the same participants independently. The intraclass correlation coefficients (ICCs) for intra-rater reliability were 0.945 (95% CI 0.918–0.964) for the M-scoliometer and 0.961 (95% CI 0.941–0.975) for the E-scoliometer; the corresponding inter-rater ICCs were 0.923 (95% CI 0.891–0.948) and 0.938 (95% CI 0.906–0.959), respectively. All ICCs exceeded 0.80, indicating good reliability for both devices. Agreement between the two devices was then examined using synchronized ATR measurements on the same participants: the two instruments showed good consistency (ICC = 0.941, 95% CI 0.910–0.962), and Bland–Altman analysis revealed a small mean difference (bias = 0.2°; 95% limits of agreement, −1.4° to +1.8°) with no evidence of proportional bias across the measured range, indicating that the two devices produced comparable ATR measurements at the individual level.

#### 2.3.2 Formal screening procedure

The screening protocol for this study was based on GB/T 16133–2014. All subjects were required to sequentially complete the following four-step standardized procedure: (1) General Visual Inspection: The subject removes shoes or wears flat slippers, stands in natural posture wearing the standardized screening garment described below, and the examiner observes from behind to check for symmetry of the shoulders, scapulae, lumbar notches, and pelvis, recording any abnormalities; (2) Adams Forward Bend Test: The subject stands with feet together, knees straight, and hands clasped, bending forward to approximately 90°. The examiner observes each segment from the thoracic to the lumbar spine at eye level; the test is considered positive if a rib hump, paraspinal prominence, or trunk asymmetry is observed; (3) ATR Measurement: In the forward-bending position, the measuring device was placed sequentially at the highest protrusions of the thoracic, thoracolumbar, and lumbar regions, and the maximum ATR value and its location were recorded. (4) Spinal Movement Test: Performed whenever any of the preceding steps was abnormal, that is, an abnormal general visual inspection, a positive Adams forward bend test, or a maximum ATR ≥ 5°. The subject slowly performed spinal flexion, extension, left and right lateral bending, and left and right axial rotation, twice each, and then resumed a natural standing posture to relax the shoulder, neck and back musculature. The general visual inspection and the ATR measurement at the thoracic, thoracolumbar and lumbar levels were then repeated using the same device. Asymmetry arising from compensatory muscular guarding resolves once the musculature is relaxed, whereas asymmetry of structural origin does not; the values obtained on this second measurement were therefore taken as the basis for the final screening classification. A schematic illustration of the four-step procedure is provided in Supplementary Figure S2. Garment conditions were standardized before screening began. The requirements were issued at a briefing convened by the local education bureau and conveyed to guardians by class teachers before the screening date: girls wore a thin wide-strap vest-style sports bra and boys a single-layer close-fitting cotton vest, both approximately 0.5 to 1 mm thick, with no additional layers permitted. The back was therefore not exposed, although the thoracic, thoracolumbar and lumbar surface landmarks remained identifiable through the single garment layer. Before both the general visual inspection and the ATR measurement, examiners smoothed out folds and seams, repositioned shoulder straps clear of the measurement sites and ensured that the hem did not obscure the lumbar region; this adjustment was specified as a fixed step in the operating manual. ATR was measured over the garment at all three spinal levels; when a participant attended in non-compliant clothing, such as a thick or multilayered garment, the back was exposed for measurement. All screening was conducted by same-sex examiners in a single closed room. The same garment requirements, adjustment procedure and operating manual applied to both device groups.

Based on the above procedure, screening results were classified into three mutually exclusive categories: no scoliosis, defined as a normal general visual inspection, a negative forward bend test and a maximum ATR < 5°; postural imbalance, defined as an abnormal general visual inspection, a positive forward bend test or an initial maximum ATR ≥ 5°, together with a maximum ATR < 5° on the second measurement; and positive screening, defined as a maximum ATR ≥ 5° on the second measurement, for which referral to a medical facility for radiographic confirmation (Cobb angle measurement) was recommended.

Both groups were assessed by trained medical personnel following an identical procedure: the M-scoliometer group using a manual scoliometer (Zhengzhou Jianheng Medical Technology Co., Ltd., China) and the E-scoliometer group using an electronic scoliometer (Jifeng Technology Co., Ltd., China); a positive cutoff of ATR ≥ 5° was applied to both groups. For both groups, the duration of the ATR measurement and a 5-point cooperation scale (see Supplementary Table S1) were recorded for each participant to allow comparison of screening efficiency and operational feasibility. Timing was confined to the ATR measurement, beginning when the device was first placed on the back and ending when the measurement was complete and the reading had been captured; the general visual inspection, the Adams forward bend test, spinal movement test and assignment of the cooperation score were not included. Timing ran continuously, so any repositioning or repeated measurement within this interval was captured. A trained recorder from the research team was assigned to every examination in both groups and was responsible for timing and data entry while the examiner performed the measurement: in the M-scoliometer group the recorder operated a stopwatch and entered the reading, whereas in the E-scoliometer group the interval was registered by the internal timer of the device and the reading was stored automatically. Transfer of the records into the study database took place after each screening session and was not timed. Positive screening results were stratified into three mutually exclusive severity grades based on the maximum ATR value (24, 25), applied identically to both groups: Grade 1 (mild), 5° ≤ ATR < 7°, with periodic follow-up screening recommended; Grade 2 (moderate), 7° ≤ ATR ≤ 10°, with referral for imaging evaluation; and Grade 3 (severe), ATR > 10°, with immediate referral to an orthopedic specialist. The primary outcome was the positive screening rate, defined as the proportion of children screening positive (maximum ATR ≥ 5°); secondary outcomes were the severity distribution, the screening time per child and the cooperation score. Radiographic confirmation took place outside the screening program and the results were not available to the research team, so no reference standard was obtained: sensitivity, specificity and positive predictive value could not be estimated, and diagnostic accuracy was not assessed. Because the devices were applied to independently allocated groups rather than to the same children, the between-group comparison concerns population-level positive screening rates.

#### 2.3.3 Demographic data collection and postural assessment

Demographic information for each participating student, including name, sex, age, and grade, was obtained by scanning school-prepared personal information QR codes, ensuring consistency with official school registration records. Height and weight were sourced from a routine school physical examination conducted concurrently with the screening, measured by trained school health personnel following standardized procedures. BMI was calculated as weight divided by height squared (kg/m^2^) and categorized into five groups using sex- and age-specific cut-off values from Chinese national standards WS/T 456–2014 and WS/T 586–2018 (26, 27); detailed criteria are provided in Supplementary Table S2.

Postural assessment was performed by the same group of trained and certified medical personnel. All indicators were assessed by visual inspection while the child stood, before the Adams forward bend test and ATR measurement, so that screening status was unknown at the time of assessment. Under field conditions, however, assessors independent of the screening team were not available, and examiners were therefore not blinded to the findings of the preceding general visual inspection. A total of seven postural indicators were evaluated: forward head posture (FHP), shoulder height asymmetry (SHA), winged scapula (WS), kyphosis, flat back (FB), anterior pelvic tilt (APT), and leg alignment (LA; classified into four categories: normal, X-type, O-type, and XO-type). The first six indicators were recorded as binary outcomes (normal/abnormal), while LA was recorded as a four-category variable. Detailed examination methods and criteria for positive findings are provided in Supplementary Table S3.

### 2.4 Questionnaire survey

Following the postural assessment, a self-designed structured questionnaire was distributed to the legal guardians of each participating student. Parents were asked to complete the questionnaire in writing on behalf of their children, and the forms were collected on the same day. The questionnaire covered a total of 10 variables related to daily life and study habits, falling into the following two categories (see Supplementary Table S4):

(1) Behavior and Lifestyle Habits: daily electronic device use; daily physical exercise; pen-holding posture; interocular difference in myopia degree; picky eating habits; habitual standing posture; mattress firmness; attendance at extracurricular classes. (2) School Learning Environment: regular adjustment of chair and desk height; regular rotation of classroom seating between left and right sides.

Before completing the questionnaire, researchers provided parents with a standardized explanation of the definitions and completion requirements for each item to ensure consistent understanding. For pen-holding posture, the child was asked to demonstrate their usual writing grip in the presence of the guardian, who then identified the matching type from an illustrated reference card showing all 10 grip types (Supplementary Figure S3). All questionnaires were checked individually by researchers; items with obvious omissions or logical inconsistencies were verified and corrected on the spot. After collection, a secondary review was conducted prior to data entry into the database to ensure data integrity.

### 2.5 Statistical analysis

Analyses were performed using SPSS 25.0 (IBM Corp., Armonk, NY, United States) and Bootstrap. Continuous variables were expressed as mean ± standard deviation and compared using the independent-samples t test or the Mann–Whitney U test; categorical variables were presented as frequency (percentage) and compared using the chi-square test or Fisher’s exact test. The Cochran–Armitage trend test was used to assess the linear trend in positive screening rates across grades. The absolute difference in positive screening rate between the two device groups was estimated as a risk difference with a 95% confidence interval obtained by Newcombe’s hybrid score method.

Factors associated with suspected scoliosis were examined within two domains: demographic characteristics and postural indicators, and daily habits and the school learning environment. Univariable binary logistic regression was performed for each candidate variable. Categories with a univariable *p* < 0.25 (28) were entered simultaneously as binary indicators into a multivariable logistic regression model for the corresponding domain (Enter method); no stepwise selection was applied at any stage. Adjusted odds ratios are reported for every entered category irrespective of statistical significance.

Because classes were the units of sampling, a joint model was then fitted across both domains using generalized estimating equations, with a binomial distribution, a logit link, an exchangeable working correlation structure, robust variance estimation and class as the clustering unit (29). The within-class correlation was estimated from an intercept-only model. The joint model comprised the factors significant in either domain-specific model together with grade, entered as binary indicators. Discrimination was assessed by the area under the receiver operating characteristic curve, calibration by the Hosmer–Lemeshow test and the Brier score, and internal validation by 1,000 bootstrap resamples to obtain an optimism-corrected AUC (30). The joint model was refitted with an independent working correlation and with clustering at the school level as sensitivity analyses.

Results are presented as odds ratios with 95% confidence intervals. A two-sided *p* < 0.05 was considered statistically significant. Analyses were based on complete cases; no values were imputed. Participant flow and the completeness of the analytic samples are reported in Supplementary Table S7.

## 3 Results

### 3.1 Overall screening results

Of 11,668 pupils enrolled in grades 1 to 5 at the 10 sampled schools, 22 were ineligible because they were unable to complete the Adams forward bend test and consent was obtained for all remaining pupils; 46 were absent on the screening day and 13 were excluded after examination because no valid ATR measurement could be obtained. A total of 11,587 pupils were therefore analyzed, including 5,655 boys and 5,932 girls (Supplementary Table S7). A total of 187 cases of suspected scoliosis were identified, resulting in an overall positive screening rate of 1.61%. The positive screening rate was higher among females than males (1.70% vs. 1.52%), but this difference was not statistically significant (*χ*^2^ = 0.494, *p* = 0.482). Among the suspected scoliosis cases, 59.4% (111/187) were classified as Grade 1 (mild), 30.5% (57/187) as Grade 2 (moderate), and 10.2% (19/187) as Grade 3 (severe). Detailed screening results by grade and gender are shown in Table 1.

**Table 1:** Overall screening characteristics of the study population.

Variable	No. screened	Suspected scoliosis cases	Percentage (%)	Positive screening rate (%)
Total	11,587	187	100	1.61
Sex				
Male	5,655	86	48.8	1.52
Female	5,932	101	51.2	1.70
Grade				
Grade 1	2,318	6	20.0	0.26
Grade 2	2,456	35	21.2	1.43
Grade 3	2,389	38	20.6	1.59
Grade 4	2,281	46	19.7	2.02
Grade 5	2,143	62	18.5	2.89

Severity classification of suspected scoliosis cases	No. of cases	Percentage (%)
Grade 1 (mild)	111	59.4
Grade 2 (moderate)	57	30.5
Grade 3 (severe)	19	10.2

Percentage (%) for Sex and Grade refers to the proportion of the total screened population in each subgroup. For Severity classification, Percentage (%) refers to the proportion of suspected scoliosis cases within each severity category. Positive screening rate (%) refers to the proportion of suspected scoliosis cases within each subgroup. Positive screening rates were calculated among all screened children. Severity grades were defined by the maximum ATR: Grade 1 (mild), 5° ≤ ATR < 7°; Grade 2 (moderate), 7° ≤ ATR ≤ 10°; Grade 3 (severe), ATR > 10°.

The positive screening rate of suspected scoliosis showed a significant upward trend with increasing grade level. The positive screening rate was lowest in Grade 1 (0.26%) and highest in Grade 5 (2.89%). The positive screening rates for grades 2–5 were 1.43, 1.59, 2.02, and 2.89%, respectively (*χ*^2^ = 51.782, *p* < 0.001). The Cochran-Armitage trend test confirmed a significant linear trend (*p* < 0.001). Figure 1 shows the grade-specific positive screening rates by sex. Both sexes followed the overall upward trend across grades, but the sex-specific rates fluctuated and their 95% confidence intervals overlapped at every grade: boys had the higher rate at Grades 1 and 4, and girls at Grades 2, 3, and 5. At Grade 5, the rate in girls (3.17%) exceeded that in boys (2.60%). Given the overlapping confidence intervals and the non-significant overall sex difference (*p* = 0.482), these grade-specific differences should be interpreted with caution.

**Figure 1:**
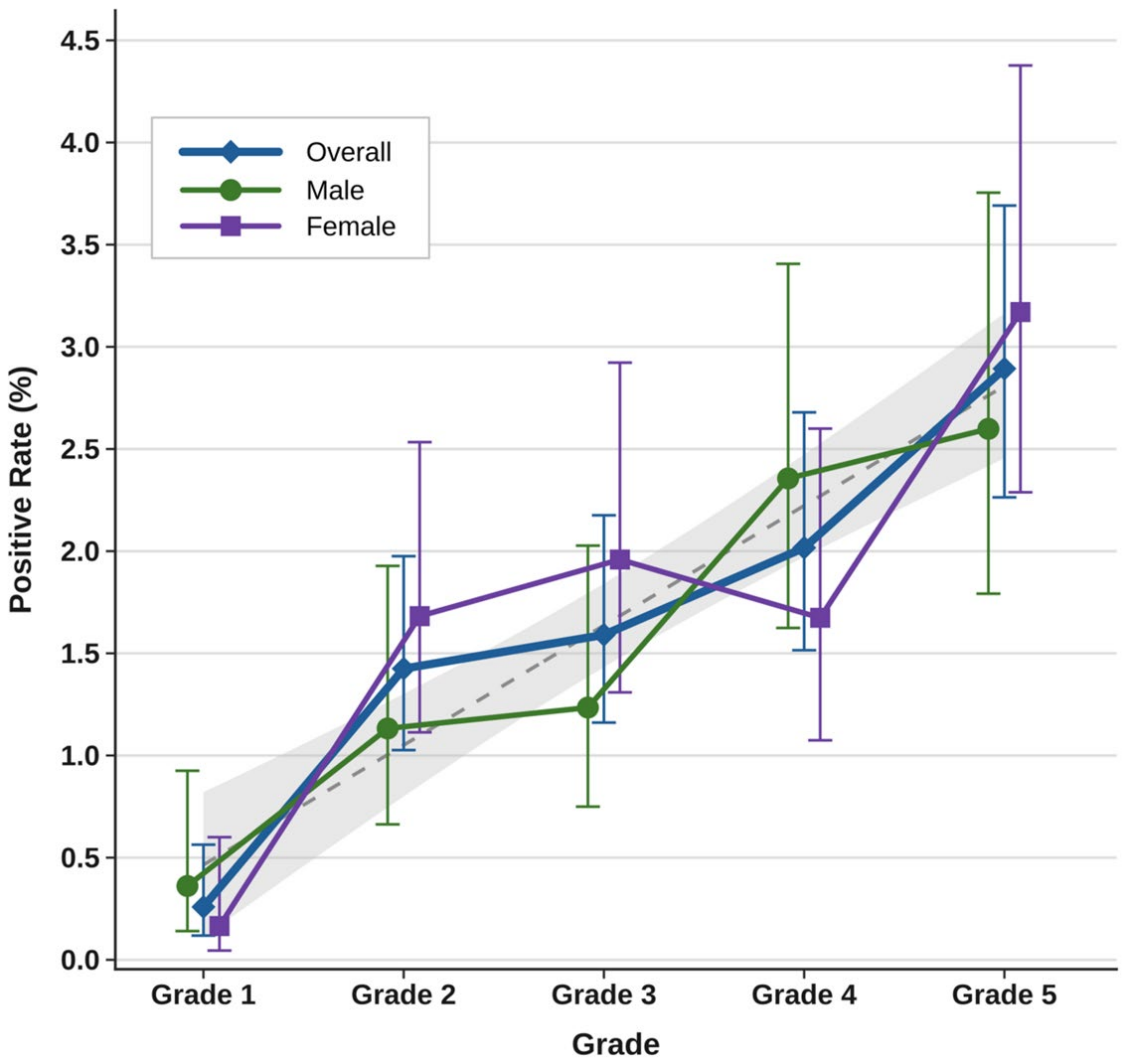
Grade-specific positive screening rates for suspected scoliosis stratified by sex. Line plots show the positive screening rates across Grades 1–5 for the overall sample and by sex (male/female). Shaded area indicates the 95% confidence band of the linear trend for the overall sample; dashed line indicates the overall linear trend. Error bars represent 95% confidence intervals.

### 3.2 Comparison of M-scoliometer and E-scoliometer screening

#### 3.2.1 Basic characteristics of the study population

This study included a total of 11,587 elementary school students (grades 1–5) from Harbin, Heilongjiang Province, comprising 5,697 participants in the M-scoliometer group and 5,890 in the E-scoliometer group. There were no significant differences between the two groups in terms of gender distribution, grade distribution, or cooperation scores, indicating good comparability. The baseline characteristics of the participants in both groups are detailed in Table 2.

**Table 2:** Comparison of baseline characteristics between the M-scoliometer group and the E-scoliometer group.

Variable	M-scoliometer group (*n* = 5,697)	E-scoliometer group (*n* = 5,890)	Statistic	*p* value
Sex, *n* (%)			*χ*^2^ = 1.684	0.194
Male	2,745 (48.2)	2,910 (49.4)		
Female	2,952 (51.8)	2,980 (50.6)		
Grade distribution, *n* (%)			*χ*^2^ = 2.296	0.681
Grade 1	1,167 (20.5)	1,151 (19.5)		
Grade 2	1,208 (21.2)	1,248 (21.2)		
Grade 3	1,178 (20.7)	1,211 (20.6)		
Grade 4	1,111 (19.5)	1,170 (19.9)		
Grade 5	1,033 (18.1)	1,110 (18.8)		
Cooperation score	4.16 ± 0.73	4.15 ± 0.72	Z = −0.884	0.377

Data are presented as n (%) or mean ± SD. M-scoliometer: manual scoliometer (traditional device); E-scoliometer: electronic scoliometer (smart screening device). Sex and grade distribution were compared using the chi-square (*χ*^2^) test. Cooperation score was compared using the Mann–Whitney U test (reported as *Z* value). *p* < 0.05 was considered statistically significant.

#### 3.2.2 Advantages of screening using smart devices

The M-scoliometer group detected 95 suspected cases of scoliosis, giving a positive screening rate of 1.67%, and the E-scoliometer group detected 92 suspected cases, giving a rate of 1.56%. The absolute difference between the two groups (M-scoliometer minus E-scoliometer) was 0.11 percentage points (95% CI − 0.36 to 0.57), and no statistically significant difference was detected (*p* = 0.706). Regarding the distribution of severity, there was also no significant difference between the two groups (*p* = 0.556). Figure 2 illustrates the variation in positive screening rates across grade levels for both groups, while Figure 3 shows the distribution of scoliosis severity by grade level.

**Figure 2:**
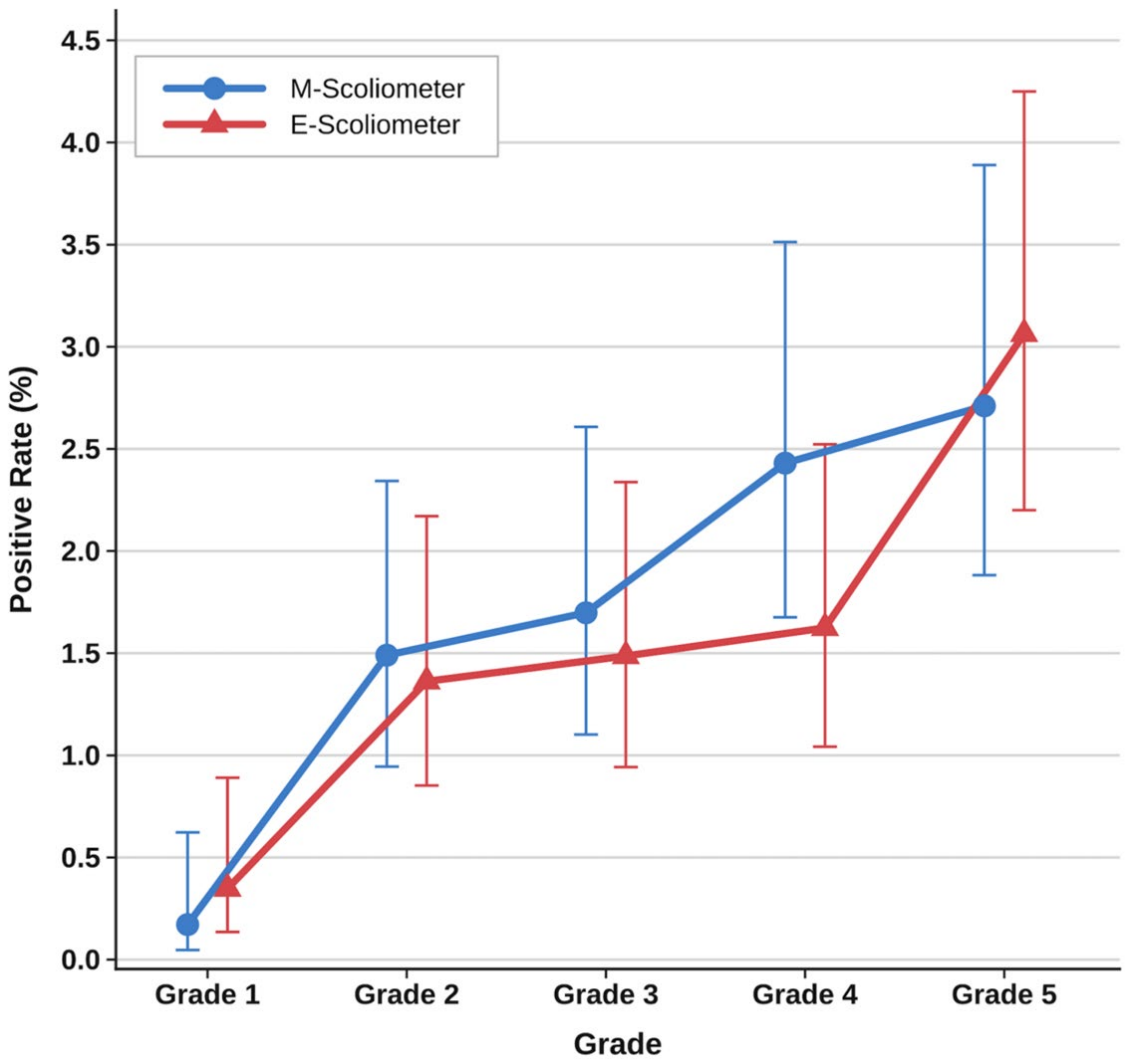
Grade-specific positive screening rates for suspected scoliosis in the M-scoliometer and E-scoliometer groups. Line plots show the grade-specific positive screening rates for suspected scoliosis across Grades 1–5 in the M-scoliometer and E-scoliometer groups. Error bars represent 95% confidence intervals. M-scoliometer: manual scoliometer; E-scoliometer: electronic scoliometer.

**Figure 3:**
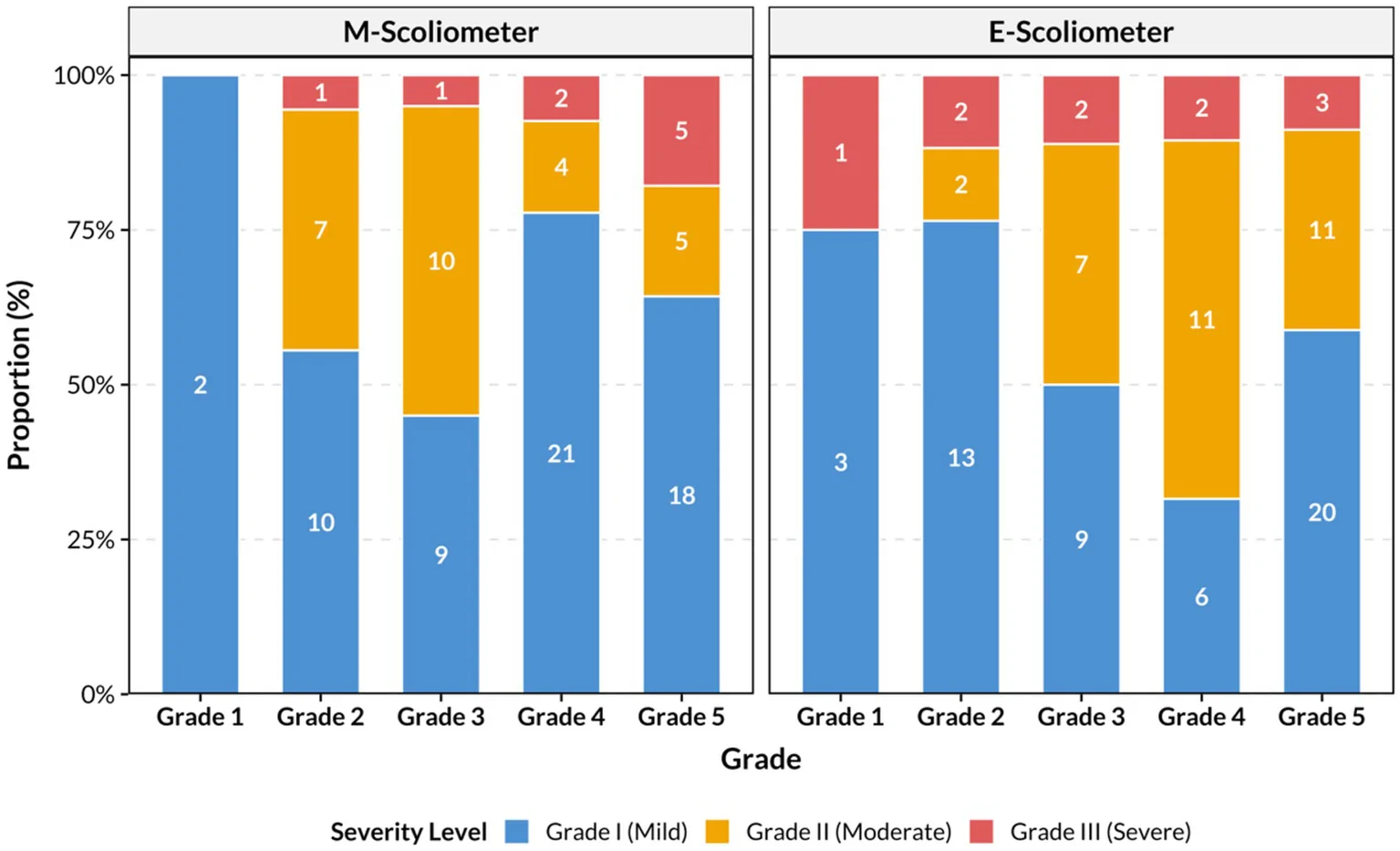
Grade-specific severity distribution of suspected scoliosis cases in the M-scoliometer and E-scoliometer groups. Severity was classified based on the maximum ATR into mutually exclusive grades: Grade 1 (mild), 5° ≤ ATR < 7°; Grade 2 (moderate), 7° ≤ ATR ≤ 10°; Grade 3 (severe), ATR > 10°. M-scoliometer: Manual scoliometer. E-scoliometer: Electronic scoliometer.

In terms of screening efficiency, the average screening time in the E-scoliometer group was significantly shorter than that in the M-scoliometer group (*p* < 0.001), with a reduction of 18.97 s, corresponding to a 54.2% shorter measurement time. This difference demonstrated a large effect size (Cohen’s d = 2.91). Details are presented in Table 3. The E-scoliometer group had a smaller standard deviation in screening time, suggesting superior operational stability of the smart device and less variability in results (Figure 4A). Regarding cooperation, there was no statistically significant difference in scores between the two groups (Figure 4B), indicating that the level of cooperation among participants was comparable, indicating that cooperation was comparable between the two groups and is therefore unlikely to account for the difference in measurement time.

**Table 3:** Comparison of screening outcomes between the M-scoliometer group and the E-scoliometer group.

Outcome indicator	M-scoliometer group (*n* = 5,697)	E-scoliometer group (*n* = 5,890)	Statistic	*p* value
Positive screening rate, % (*n*)	1.67 (95)	1.56 (92)	*χ*^2^ = 0.142	0.706
Screening time, seconds	35.00 ± 8.19	16.04 ± 4.22	*t* = 157.3	<0.001
Severity distribution of suspected scoliosis cases, *n* (%)			*χ*^2^ = 1.173	0.556
Grade 1 (mild)	60 (63.2)	51 (55.4)		
Grade 2 (moderate)	26 (27.4)	31 (33.7)		
Grade 3 (severe)	9 (9.5)	10 (10.9)		

Data are presented as % (n) or mean ± SD. M-scoliometer: manual scoliometer (traditional device); E-scoliometer: electronic scoliometer (smart screening device). Positive screening rate and severity distribution were compared using the chi-square (χ^2^) test. Screening time was compared using the independent-samples t-test. Severity was classified based on the maximum ATR into mutually exclusive grades: Grade 1 (mild), 5° ≤ ATR < 7°; Grade 2 (moderate), 7° ≤ ATR ≤ 10°; Grade 3 (severe), ATR > 10°. *p* < 0.05 was considered statistically significant.

**Figure 4:**
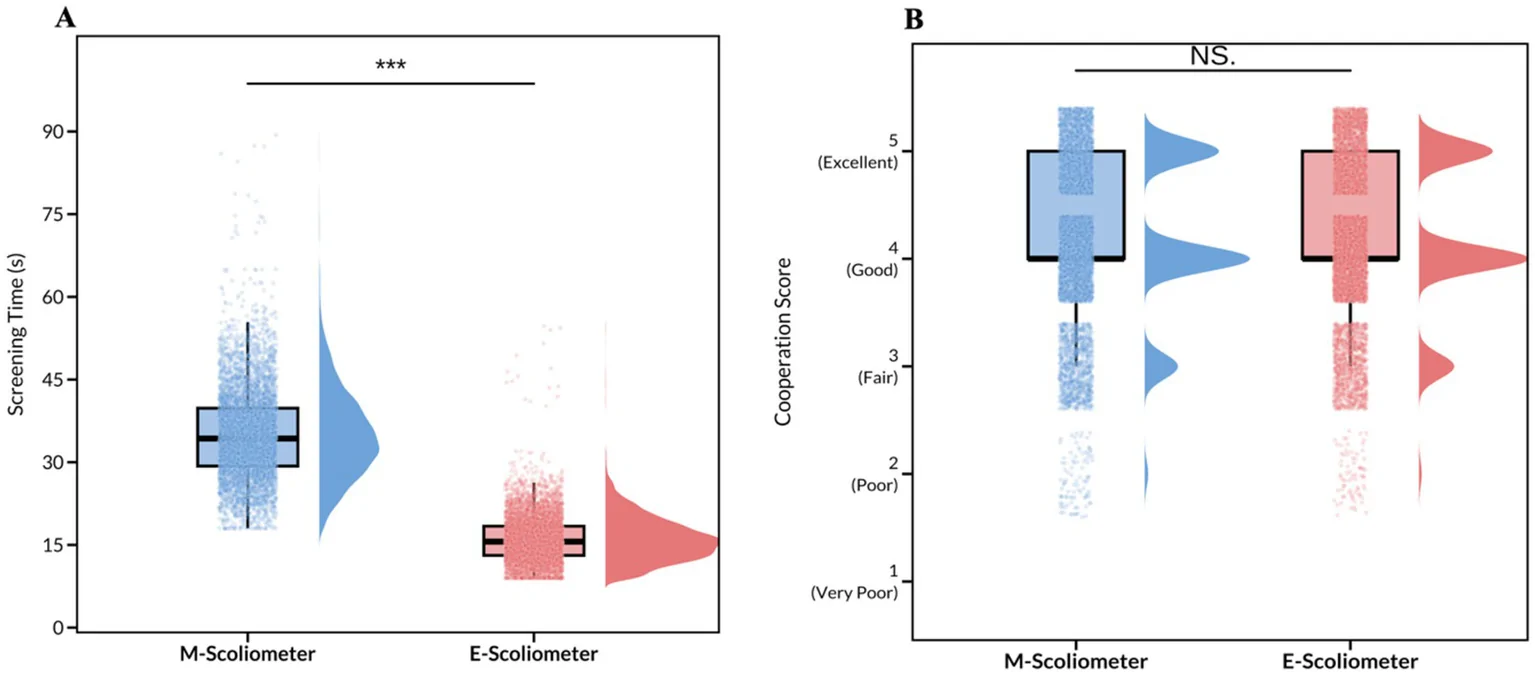
Distribution of screening time and cooperation scores in the M-scoliometer and E-scoliometer groups. Raincloud plots display raw data points, box plots, and kernel density curves. **(A)** Screening time (seconds) by group. **(B)** Cooperation score by group. M-scoliometer: Manual scoliometer. E-scoliometer: Electronic scoliometer.

### 3.3 Risk factors for suspected scoliosis

#### 3.3.1 Demographic characteristics and poor posture

##### 3.3.1.1 Comparison of demographics and posture between groups

Two classes were sampled from each grade in every school, yielding 100 classes and 3,674 pupils within the M-scoliometer analytic set; 3 did not complete the postural assessment and 71 returned questionnaires that were unusable on verification, leaving 3,600 pupils for the risk-factor analysis. All variables were complete for these 3,600 pupils (Supplementary Table S7). Among them, 59 cases (1.64%) were identified as suspected scoliosis, while 3,541 cases (98.36%) were in the normal group. There was no statistically significant difference in gender distribution between the two groups (*p* = 0.224). Fifth-grade students accounted for the highest proportion in the scoliosis group, at 19/59 (32.2%); the difference in grade distribution was statistically significant (*p* = 0.018). Students in the scoliosis group were significantly taller than those in the normal group (*p* = 0.001), while there were no statistically significant differences in weight or BMI between the two groups. Regarding BMI classification, the proportion of students classified as mild wasting was significantly higher in the suspected scoliosis group than in the normal group (13.6% vs. 3.8%), and the overall distribution of BMI categories differed significantly between the two groups (*p* = 0.002). In terms of posture, the incidence of FHP, SHA, kyphosis, and FB was significantly higher in the scoliosis group than in the normal group (*p* < 0.05). There were no statistically significant differences in WS, APT, and LA between the two groups (Table 4).

**Table 4:** Comparison of demographic characteristics and postural indicators between the normal group and the suspected scoliosis group.

Variable	Normal group (*n* = 3,541)	Suspected scoliosis group (*n* = 59)	Statistic	*p* value
Sex, *n* (%)			*χ*^2^ = 1.477	0.224
Male	1,693 (47.8)	23 (39.0)		
Female	1848 (52.2)	36 (61.0)		
Grade distribution, *n* (%)			*χ*^2^ = 11.959	0.018
Grade 1	717 (20.2)	3 (5.1)		
Grade 2	710 (20.1)	10 (16.9)		
Grade 3	707 (20.0)	13 (22.0)		
Grade 4	706 (19.9)	14 (23.7)		
Grade 5	701 (19.8)	19 (32.2)		
Height, cm	130.44 ± 8.84	134.42 ± 8.20	*t* = −3.427	0.001
Weight, kg	27.58 ± 5.83	28.64 ± 5.60	*t* = −1.380	0.168
BMI	16.10 ± 2.34	15.77 ± 2.23	*t* = 1.069	0.285
BMI category, *n* (%)			*χ*^2^ = 16.558	0.002
Normal weight	2,486 (70.2)	34 (57.6)		
Mild wasting	136 (3.8)	8 (13.6)		
Moderate to severe wasting	52 (1.5)	2 (3.4)		
Overweight	496 (14.0)	8 (13.6)		
Obesity	371 (10.5)	7 (11.9)		
Forward head posture (FHP), *n* (%)			*χ*^2^ = 7.811	0.005
No	2,382 (67.3)	29 (49.2)		
Yes	1,159 (32.7)	30 (50.8)		
Shoulder height asymmetry (SHA), *n* (%)			*χ*^2^ = 23.433	<0.001
No	2,256 (63.7)	19 (32.2)		
Yes	1,285 (36.3)	40 (67.8)		
Winged scapula (WS), *n* (%)			*χ*^2^ = 0.005	0.945
No	2,864 (80.9)	47 (79.7)		
Yes	677 (19.1)	12 (20.3)		
Kyphosis, *n* (%)			*χ*^2^ = 11.159	<0.001
No	2,534 (71.6)	30 (50.8)		
Yes	1,007 (28.4)	29 (49.2)		
Flat back (FB), n (%)			*χ*^2^ = 62.132	<0.001
No	2,654 (75.0)	17 (28.8)		
Yes	887 (25.0)	42 (71.2)		
Anterior pelvic tilt (APT), n (%)			*χ*^2^ = 0.028	0.867
No	2,697 (76.2)	46 (78.0)		
Yes	844 (23.8)	13 (22.0)		
Leg alignment (LA), *n* (%)			*χ*^2^ = 2.076	0.557
Normal	3,109 (87.8)	54 (91.5)		
X-type	248 (7.0)	2 (3.4)		
O-type	141 (4.0)	3 (5.1)		
XO-type	43 (1.2)	0 (0.0)		

Data are presented as *n* (%) or mean ± SD. BMI, body mass index; FHP, forward head posture; SHA, shoulder height asymmetry; WS, winged scapula; FB, flat back; APT, anterior pelvic tilt; LA, leg alignment. Sex, grade distribution, BMI category, FHP, SHA, WS, kyphosis, FB, APT, and LA were compared using the chi-square (*χ*^2^) test. Height, weight, and BMI were compared using independent-samples *t*-tests.

##### 3.3.1.2 Univariate and multivariate logistic regression analysis

Univariate analysis revealed that grade, height, weight, sex (female), BMI category (mild wasting, moderate to severe wasting), FHP, SHA, kyphosis, and FB all had *p*-values < 0.25 and were therefore included in the multivariate logistic regression analysis.

In the multivariable model for this domain, mild wasting (OR = 4.930, 95% CI 1.943–12.511, *p* = 0.001), SHA (OR = 3.768, 95% CI 1.721–8.253, *p* < 0.001) and FB (OR = 7.227, 95% CI 3.639–14.351, *p* < 0.001) remained significantly associated with suspected scoliosis, with FB showing the strongest association (Table 5). Because this model contained demographic and postural variables only, these estimates are mutually adjusted within, but not across, domains; estimates that are additionally adjusted for lifestyle and learning-environment factors and for classroom-level clustering are presented in Section 3.3.3.

**Table 5:** Univariate and multivariate logistic regression analysis of demographic characteristics and postural indicators associated with suspected scoliosis.

Variable	Category	Univariate analysis		Multivariate analysis	
		*p* value	OR (95% CI)	*p* value	OR (95% CI)
Grade		0.001	1.378 (1.151–1.689)	0.107	1.461 (0.919–2.309)
Height, cm		<0.001	1.608 (1.255–2.058)	0.895	0.957 (0.508–1.602)
Weight, kg		0.141	1.159 (0.950–1.398)	0.747	1.076 (0.673–1.656)
BMI		0.277	0.941 (0.840–1.047)	—	—
Sex	Male		1		1
	Female	0.191	1.392 (0.884–2.264)	0.194	1.390 (0.831–2.363)
BMI category	Normal weight		1		1
	Mild wasting	<0.001	4.301 (1.953–9.470)	0.001	4.930 (1.943–12.511)
	Moderate to severe wasting	0.163	2.812 (0.658–12.016)	0.553	1.883 (0.232–15.256)
	Overweight	0.677	1.179 (0.543–2.563)	—	—
	Obesity	0.442	1.380 (0.607–3.135)	—	—
Forward head posture (FHP)	No		1		1
	Yes	0.005	2.123 (1.268–3.553)	0.061	2.326 (0.963–5.620)
Shoulder height asymmetry (SHA)	No		1		1
	Yes	<0.001	3.690 (2.128–6.398)	<0.001	3.768 (1.721–8.253)
Winged scapula (WS)	No		1		1
	Yes	0.945	1.098 (0.579–2.082)	—	—
Kyphosis	No		1		1
	Yes	<0.001	2.433 (1.453–4.074)	0.086	2.362 (0.887–6.295)
Flat back (FB)	No		1		1
	Yes	<0.001	7.348 (4.162–12.974)	<0.001	7.227 (3.639–14.351)
Anterior pelvic tilt (APT)	No		1		1
	Yes	0.867	0.913 (0.491–1.698)	—	—
Leg alignment (LA)	Normal		1		1
	X-type	0.409	0.466 (0.113–1.919)	—	—
	O-type	0.925	1.303 (0.403–4.214)	—	—

OR, odds ratio; CI, confidence interval; BMI, body mass index; FHP, forward head posture; SHA, shoulder height asymmetry; WS, winged scapula; FB, flat back; APT, anterior pelvic tilt; LA, leg alignment. “—” indicates categories excluded from multivariate analysis (*p* ≥ 0.25 in univariate analysis or zero events in the suspected scoliosis group). All categories with *p* < 0.25 in univariate analysis were entered simultaneously (Enter method); no stepwise selection was applied. Each retained category was modeled as a binary indicator versus all remaining categories combined. Estimates are adjusted within, but not across, domains (see Table 8). *p* < 0.05 was considered statistically significant.

#### 3.3.2 Lifestyle and learning habits

##### 3.3.2.1 Comparison of lifestyle and study habits between groups

The results of the chi-square test regarding daily life and study habits showed that, compared to the normal group, the suspected scoliosis group spent less time on daily physical exercise and had a higher proportion of students with picky eating habits (*p* < 0.001). However, compared to the suspected scoliosis group, the normal group was more likely to regularly adjust their chair and desk height and regularly rotate classroom seating between left and right sides during class (*p* < 0.001). The remaining factors showed differences in the proportion of participants between the normal and suspected scoliosis groups, but none reached statistical significance (*p* > 0.05; Table 6).

**Table 6:** Comparison of daily habits and school learning environment between the normal group and the suspected scoliosis group.

Variable	Normal group (*n* = 3,541)	Suspected scoliosis group (*n* = 59)	Statistic	*p* value
Daily electronic device use, *n* (%)			*χ*^2^ = 3.644	0.162
< 1 h per day	1727 (48.8)	22 (37.3)		
1–2 h per day	1,227 (34.7)	26 (44.1)		
> 2 h per day	587 (16.6)	11 (18.6)		
Daily physical exercise, *n* (%)			*χ*^2^ = 27.260	<0.001
< 1 h per day	1,300 (36.7)	41 (69.5)		
1–2 h per day	1,607 (45.4)	15 (25.4)		
> 2 h per day	634 (17.9)	3 (5.1)		
Pen-holding posture, *n* (%)			*χ*^2^ = 12.355	0.194
Type 0 (correct grip)	1,483 (41.9)	19 (32.2)		
Type 1	579 (16.4)	8 (13.6)		
Type 2	419 (11.8)	8 (13.6)		
Type 3	271 (7.7)	9 (15.3)		
Type 4	198 (5.6)	6 (10.2)		
Type 5	311 (8.8)	3 (5.1)		
Type 6	162 (4.6)	5 (8.5)		
Type 7	44 (1.2)	0 (0.0)		
Type 8	43 (1.2)	1 (1.7)		
Type 9	31 (0.9)	0 (0.0)		
Interocular difference in myopia degree ≥ 100° (1.00 diopter), *n* (%)			*χ*^2^ = 1.725	0.189
No	3,061 (86.4)	47 (79.7)		
Yes	480 (13.6)	12 (20.3)		
Picky eating habits, *n* (%)			*χ*^2^ = 25.165	<0.001
No	2,664 (75.2)	27 (45.8)		
Yes	877 (24.8)	32 (54.2)		
Habitual standing posture, *n* (%)			*χ*^2^ = 0.961	0.619
Always stands with good posture	645 (18.2)	12 (20.3)		
Occasionally shifts or adjusts stance	1728 (48.8)	25 (42.4)		
Stands however feels comfortable	1,168 (33.0)	22 (37.3)		
Mattress firmness, *n* (%)			*χ*^2^ = 2.208	0.332
Medium	2,623 (74.1)	41 (69.5)		
Soft	593 (16.7)	14 (23.7)		
Firm	325 (9.2)	4 (6.8)		
Extracurricular class attendance, *n* (%)			*χ*^2^ = 1.186	0.757
None	653 (18.4)	13 (22.0)		
Academic or arts classes only	1,473 (41.6)	21 (35.6)		
Sports or physical activity only	569 (16.1)	9 (15.3)		
Both academic and sports classes	846 (23.9)	16 (27.1)		
Regular adjustment of chair and desk height, *n* (%)			*χ*^2^ = 41.320	<0.001
No	1,239 (35.0)	45 (76.3)		
Yes	2,302 (65.0)	14 (23.7)		
Regular rotation of classroom seating between left and right sides, *n* (%)			*χ*^2^ = 29.683	<0.001
No	1,374 (38.8)	44 (74.6)		
Yes	2,167 (61.2)	15 (25.4)		

Data are presented as *n* (%). All variables were compared using the chi-square (*χ*^2^) test. Pen-holding posture types 0–9 are classified according to the standardized grading system used in this study (see Supplementary Table S4; Supplementary Figure S3). *p* < 0.05 was considered statistically significant.

##### 3.3.2.2 Univariate and multivariate logistic regression analysis

Univariate analysis revealed that daily electronic device use of 1–2 h and >2 h, daily physical exercise <1 h, pen-holding posture types 3 and 4, interocular difference in myopia degree ≥ 100° (1.00 diopter), picky eating, regular adjustment of chair and desk height, soft mattresses, and regular rotation of classroom seating between left and right sides all had *p*-values < 0.25 and were included in the multivariate analysis.

In the multivariable model for this domain, daily physical exercise < 1 h per day (OR = 3.917, 95% CI 2.241–6.846, *p* < 0.001), thumb-inward grip pen-holding posture (Type 3) (OR = 2.137, 95% CI 1.076–4.243, *p* = 0.042) and picky eating (OR = 3.563, 95% CI 1.794–7.076, *p* < 0.001) were associated with higher odds of suspected scoliosis, whereas regular adjustment of chair and desk height (OR = 0.157, 95% CI 0.083–0.297, *p* < 0.001) and regular rotation of classroom seating between left and right sides (OR = 0.204, 95% CI 0.109–0.382, *p* < 0.001) were associated with lower odds (Table 7). These estimates are likewise mutually adjusted within, but not across, domains.

**Table 7:** Univariate and multivariate logistic regression analysis of daily habits and school learning environment associated with suspected scoliosis.

Variable	Category	Univariate analysis		Multivariate analysis	
		*p* value	OR (95% CI)	*p* value	OR (95% CI)
Daily electronic device use	< 1 h per day		1		1
	1–2 h per day	0.185	1.299 (0.883–1.912)	0.286	1.703 (0.639–4.539)
	> 2 h per day	0.217	1.439 (0.796–2.601)	0.294	1.690 (0.634–4.503)
Daily physical exercise	> 2 h per day		1		1
	< 1 h per day	<0.001	6.820 (3.434–13.542)	<0.001	3.917 (2.241–6.846)
	1–2 h per day	0.407	1.973 (0.391–9.829)	—	—
Pen-holding posture	Type 0 (correct grip)		1		1
	Type 1	0.691	0.804 (0.380–1.704)	—	—
	Type 2	0.839	1.177 (0.555–2.497)	—	—
	Type 3	0.034	2.185 (1.063–4.490)	0.042	2.137 (1.076–4.243)
	Type 4	0.123	1.960 (0.833–4.615)	0.109	2.229 (0.836–5.938)
	Type 5	0.444	0.555 (0.173–1.784)	—	—
	Type 6	0.271	1.930 (0.602–6.223)	—	—
	Type 8	1.000	0.976 (0.132–7.205)	—	—
Interocular difference in myopia degree ≥ 100°	No		1		1
	Yes	0.189	1.679 (0.773–3.639)	0.322	1.412 (0.714–2.795)
Picky eating habits	No		1		1
	Yes	<0.001	3.565 (2.124–5.984)	<0.001	3.563 (1.794–7.076)
Regular adjustment of chair and desk height	No		1		1
	Yes	<0.001	0.167 (0.092–0.306)	<0.001	0.157 (0.083–0.297)
Habitual standing posture	Always stands with good posture		1		1
	Occasionally shifts or adjusts stance	0.327	0.771 (0.458–1.298)	—	—
	Stands however feels comfortable	0.486	1.208 (0.709–2.057)	—	—
Mattress firmness	Medium		1		1
	Soft	0.155	1.547 (0.844–2.836)	0.103	1.703 (0.898–3.232)
	Firm	0.526	0.720 (0.259–1.999)	—	—
Extracurricular class attendance	None		1		1
	Academic or arts classes only	0.353	0.776 (0.453–1.328)	—	—
	Sports or physical activity only	0.866	0.940 (0.460–1.923)	—	—
	Both academic and sports classes	0.565	1.185 (0.664–2.115)	—	—
Regular rotation of classroom seating between left and right sides	No		1		1
	Yes	<0.001	0.216 (0.120–0.389)	<0.001	0.204 (0.109–0.382)

OR, odds ratio; CI, confidence interval. “—” indicates categories excluded from multivariate analysis (*p* ≥ 0.25 in univariate analysis or zero events in the suspected scoliosis group). All categories with *p* < 0.25 in univariate analysis were entered simultaneously (Enter method); no stepwise selection was applied. Each retained category was modeled as a binary indicator versus all remaining categories combined. Estimates are adjusted within, but not across, domains (see Table 8). *p* < 0.05 was considered statistically significant.

#### 3.3.3 Joint model across domains with adjustment for classroom-level clustering

The joint GEE model comprised nine variables and was fitted in 3,600 pupils from 100 classes, among whom 59 were screening-positive, giving 6.56 events per variable (the parameters estimated and events per variable for all multivariable models are given in Supplementary Table S6). The within-class correlation parameter estimated from an intercept-only model was 0.049.

After mutual adjustment across domains and adjustment for within-class correlation, seven of the eight factors identified in the domain-specific models remained significantly associated with suspected scoliosis, with the direction and approximate magnitude of their earlier estimates preserved (Table 8). Flat back showed the strongest association (OR = 7.36, 95% CI 4.37–12.40, *p* < 0.001), followed by picky eating (OR = 4.19, 95% CI 2.38–7.38, *p* < 0.001), daily physical exercise < 1 h per day (OR = 3.86, 95% CI 2.26–6.59, *p* < 0.001), mild wasting (OR = 3.80, 95% CI 1.53–9.42, *p* = 0.004) and shoulder height asymmetry (OR = 3.06, 95% CI 1.71–5.48, *p* < 0.001). Regular adjustment of chair and desk height (OR = 0.18, 95% CI 0.10–0.33, *p* < 0.001) and regular rotation of classroom seating between left and right sides (OR = 0.19, 95% CI 0.10–0.37, *p* < 0.001) were associated with lower odds. Grade was associated with higher odds of suspected scoliosis (OR = 1.43 per one-grade increment, 95% CI 1.19–1.72, *p* < 0.001).

**Table 8:** Joint model of factors associated with suspected scoliosis, with mutual adjustment across domains and adjustment for classroom-level clustering.

Variable	Domain-specific modelOR (95% CI), *p*	Joint logistic modelOR (95% CI), *p*	Joint GEE modelOR (95% CI), *p*
Grade (per one-grade increment)	1.46 (0.92–2.31), 0.107	1.41 (1.13–1.75), 0.002	1.43 (1.19–1.72), <0.001
Mild wasting	4.93 (1.94–12.51), 0.001	3.87 (1.58–9.46), 0.003	3.80 (1.53–9.42), 0.004
Shoulder height asymmetry	3.77 (1.72–8.25), <0.001	3.00 (1.65–5.45), <0.001	3.06 (1.71–5.48), <0.001
Flat back	7.23 (3.64–14.35), <0.001	7.39 (4.02–13.60), <0.001	7.36 (4.37–12.40), <0.001
Daily physical exercise < 1 h/day	3.92 (2.24–6.85), <0.001	3.85 (2.11–7.05), <0.001	3.86 (2.26–6.59), <0.001
Thumb-inward pen grip (Type 3)	2.14 (1.08–4.24), 0.042	1.49 (0.62–3.55), 0.371	1.55 (0.77–3.11), 0.221
Picky eating	3.56 (1.79–7.08), <0.001	4.01 (2.25–7.16), <0.001	4.19 (2.38–7.38), <0.001
Regular adjustment of chair and desk height	0.16 (0.08–0.30), <0.001	0.17 (0.09–0.33), <0.001	0.18 (0.10–0.33), <0.001
Regular rotation of classroom seating	0.20 (0.11–0.38), <0.001	0.19 (0.10–0.36), <0.001	0.19 (0.10–0.37), <0.001

OR, odds ratio; CI, confidence interval; GEE, generalized estimating equation. Domain-specific estimates are reproduced from Tables 5, 7, in which the two domains were modeled separately. Both joint models contain all nine variables simultaneously; the joint GEE model additionally accounts for within-class correlation (exchangeable working correlation, robust variance estimation, 100 classes). All variables were entered as binary indicators, each contrasted with all remaining categories combined (for example, daily physical exercise < 1 h/day versus ≥ 1 h/day). Sensitivity analyses are presented in Supplementary Table S5, *p* < 0.05 was considered statistically significant.

Thumb-inward grip pen-holding posture (Type 3) was the only factor whose association did not persist (OR = 1.55, 95% CI 0.77–3.11, *p* = 0.221), compared with OR = 2.137 (95% CI 1.076–4.243, *p* = 0.042) in the domain-specific model. A similar estimate was obtained when the same nine variables were fitted in an ordinary logistic model without adjustment for clustering (OR = 1.49, 95% CI 0.62–3.55, *p* = 0.371).

The joint model showed good discrimination (AUC = 0.915) and adequate calibration (Hosmer–Lemeshow *χ*^2^ = 2.77, *p* = 0.948; Brier score 0.0129, versus 0.0161 for a non-informative model). Internal validation with 1,000 bootstrap resamples yielded an optimism of 0.005 and an optimism-corrected AUC of 0.910. Estimates were consistent under an independent working correlation with cluster-robust standard errors and under clustering at the school level, with all seven factors retaining *p* < 0.05 in every specification and Type 3 pen grip remaining non-significant throughout (Supplementary Table S5).

## 4 Discussion

### 4.1 Overall positive screening rate and trends by grade level

This study screened a total of 11,587 students in grades 1 through 5 from 10 public elementary schools in Harbin. The overall positive screening rate for suspected scoliosis was 1.61%, which falls within the range reported in Chinese literature on elementary school students during the same period (7, 31). The sample in this study focused on children aged 6–12, which is younger than the 6–18 age range covered by most existing studies (8, 15, 31). This partly explains why the overall positive screening rate was lower than that observed among Chinese middle school students. Specifically, younger students have not yet entered their pubertal growth spurt, and their spinal curves are relatively stable from a biomechanical perspective (32). Furthermore, epidemiological data on scoliosis among elementary school students in Northeast China are lacking. This study is therefore of significant importance in filling this data gap.

The positive screening rate showed a significant linear increase with grade level (*p* < 0.001), rising from 0.26% in grade 1 to 2.89% in grade 5, consistent with the pattern that scoliosis prevalence increases with age (7, 8, 15, 31). The biological mechanism is closely related to rapid skeletal growth during pre-adolescence. Students in grades 4–5 are often in the pre-pubertal phase of accelerated growth, characterized by a high organic matrix content in the vertebral bone and strong skeletal plasticity. If neuromuscular control of the paraspinal muscles is insufficient, this renders these students highly susceptible to rotational displacement under asymmetric loading (20). Notably, the overall sex difference was not statistically significant (*p* = 0.482), and the sex-specific rates fluctuated across grades with overlapping confidence intervals, so a consistent sex difference could not be established in this age range. The higher rate in girls than boys at Grade 5 (3.17% vs. 2.60%) is consistent with the known female predominance of adolescent idiopathic scoliosis and with girls entering puberty earlier than boys (6, 33); however, given the small and non-significant differences, this finding is hypothesis-generating and warrants confirmation in larger and older samples.

### 4.2 Comparable positive screening rates and efficiency advantages of smart devices compared with manual screening

One of the most significant innovations of this study lies in the direct comparison of the performance of the M-scoliometer and the E-scoliometer in real-world school screening settings. No statistically significant difference was detected between the two groups in the positive screening rate of suspected scoliosis (difference 0.11 percentage points, 95% CI − 0.36 to 0.57; *p* = 0.706) or in the severity distribution (*p* = 0.556), indicating that the two devices produced comparable screening yields under real-world conditions. As the study was not designed as a formal equivalence trial, this finding indicates the absence of a detected difference rather than demonstrated equivalence. This aligns with the findings of an exploratory study conducted by Zhu et al. among 27,532 students in Linxia, Gansu: there was no significant difference in the initial screening positivity rate between the M-scoliometer and the E-scoliometer (*p* = 0.657) (19).

Regarding screening efficiency, the average screening time per person in the E-scoliometer group (16.04 s) was approximately 54.2% shorter than that in the M-scoliometer group (35.00 s), with a large effect size (Cohen’s d = 2.91). Because only the ATR measurement was timed, this difference cannot originate elsewhere in the screening workflow; it reflects the combined effect of obtaining and capturing the reading, which the manual procedure required the examiner to read and the recorder to enter, whereas the electronic device registered and stored it automatically. Moreover, the E-scoliometer group showed a smaller standard deviation in screening time, indicating superior operational stability. Zhu et al. similarly confirmed that the E-scoliometer screening speed was significantly faster than that of the M-scoliometer (19.19 s vs. 36.53 s) (19). The E-scoliometer recorded ATR values and curvature direction automatically, reducing the scope for transcription error inherent in manual recording. This is of significant value in practical scenarios where large-scale screening tasks must be completed within a limited number of working days.

### 4.3 Association between demographic characteristics and poor posture and suspected scoliosis

Within the demographic and postural domain, FB (OR = 7.227), mild wasting (OR = 4.930) and SHA (OR = 3.768) showed the strongest associations with suspected scoliosis, and all three remained significantly associated in the joint model after mutual adjustment for lifestyle and learning-environment factors and after adjustment for classroom-level clustering (FB, OR = 7.36; mild wasting, OR = 3.80; SHA, OR = 3.06; all *p* ≤ 0.004; Table 8).

In this study, FB showed the strongest association among the postural indicators examined, consistent with the findings of Schlösser et al. (34), who noted that a reduction in sagittal thoracic kyphosis is commonly observed in patients with mild AIS and precedes significant progression of the coronal curve, suggesting it is an early morphological feature of scoliosis development. An analysis by Mak et al. of 1,054 AIS patients also indicated that reduced thoracic kyphosis is a consistent sagittal plane feature across all curve types, independent of the magnitude of the coronal curve (35). From a biomechanical perspective, reduced thoracic kyphosis decreases the spine’s sagittal plane anti-rotational stability, leading to increased posterior shear forces, which in turn accelerate three-dimensional rotational displacement of the vertebrae and hasten the progression of scoliosis (36). Due to the high skeletal plasticity in young children, insufficient sagittal support renders these students more susceptible to lateral rotational displacement under asymmetric loading — a mechanism particularly prominent in the elementary school population. It is worth noting that Chen et al. reported a negative association between FB and suspected scoliosis (OR = 0.37) in adolescents aged 10–13 years (37), which contradicts the findings of this study. This discrepancy may be attributed to differences in sample age, spinal maturation levels, and heterogeneity in FB assessment criteria, suggesting that this conclusion may be influenced by age group and evaluation methods. Further research is required to confirm these findings.

SHA showed the second strongest association among the postural indicators examined. Shoulder asymmetry is one of the most visually recognizable external signs of scoliosis; it may appear earlier than other clinical signs and is therefore often used as a key indicator in school-based visual screenings and home self-checks (38). Previous studies have shown that shoulder asymmetry is the most common postural abnormality among Chinese children and adolescents (39), and its severity is associated with the coronal plane Cobb angle in AIS (20). The pathophysiological mechanism of SHA is primarily related to persistent asymmetric tension in the paraspinal muscle groups following rotation of the cervical and upper thoracic spine, a mechanism that is particularly pronounced during the growth and development phase. The results of this study suggest that SHA may be a useful indicator in routine postural observations in schools, although its value as a warning sign was not evaluated against a diagnostic reference standard.

Numerous studies have consistently identified mild wasting as an independent risk factor (6, 40). This may be because children with low BMI often have insufficient intake of growth-related nutrients: calcium and vitamin D deficiencies directly weaken the skeleton’s resistance to deformation, while inadequate protein intake impairs paraspinal muscle development by inhibiting insulin-like growth factor-1 (IGF-1) production, thereby compromising the spine’s active stabilizing function (41, 42). Therefore, mild wasting may warrant attention as a monitoring target in early screening strategies for scoliosis. Whether strengthening nutritional assessment and targeted intervention reduces the occurrence or progression of scoliosis was not examined in this study and requires prospective evaluation.

### 4.4 Risk and protective factors related to daily life and study habits

Within the domain of daily habits and the school learning environment, insufficient daily physical activity and picky eating were associated with higher odds of suspected scoliosis, whereas regular adjustment of chair and desk height and regular rotation of classroom seating between left and right sides were associated with lower odds; all four associations persisted in the joint model after mutual adjustment across domains and after adjustment for classroom-level clustering (Table 8). The association initially observed for thumb-inward grip pen-holding posture did not persist under mutual adjustment and is discussed below as an exploratory finding.

Daily physical activity of less than 1 h showed the strongest association among the behavioral factors examined (OR = 3.917 in the domain-specific model; OR = 3.86 in the joint model). Because only the “< 1 hour per day” category met the univariable screening threshold, this contrast is against ≥ 1 h of daily activity combined, and the present data therefore cannot establish a graded dose–response relationship across the three activity categories. Both the 2020 World Health Organization (WHO) Physical Activity Guidelines and the Chinese Physical Activity Guidelines for Children and Adolescents explicitly recommend that children and adolescents accumulate at least 60 min of moderate-to-vigorous physical activity daily (43). A systematic review by Guan et al., which included 77,813 adolescents, confirmed a significant association between physical activity and the risk of AIS, identifying “1 hour per day” as the key threshold for a protective effect (44). Other studies have similarly confirmed that insufficient physical activity is a risk factor for scoliosis (45, 46). The mechanism is that regular exercise helps strengthen core muscles, improve paraspinal muscle symmetry, and enhance neuromuscular coordination, thereby providing active stabilizing support for the spine during the growth and development period (47). Additionally, exercise training enhances proprioceptive function and further optimizes neuromuscular coordination, thereby improving the spine’s stabilizing mechanisms during dynamic activities (48, 49). Given the current reality of widespread insufficient physical activity among elementary school students in China, ensuring 1 h of daily physical activity should be a policy priority in school spinal health management.

The association between picky eating and suspected scoliosis is one of the novel findings of this study, and it persisted after mutual adjustment for mild wasting and the remaining factors in the joint model (OR = 3.563 in the domain-specific model; OR = 4.19, 95% CI 2.38–7.38, in the joint model); there are currently no relevant reports in the international literature, making this finding of significant exploratory value. The underlying mechanism may be that children with picky eating habits often have imbalanced intake of key nutrients essential for skeletal and muscular development, such as calcium, vitamin D, and protein (41, 50, 51). Long-term nutritional deficiency can lead to reduced bone mineralization and delayed development of the paraspinal muscle groups, thereby diminishing the spine’s ability to resist lateral forces during periods of accelerated growth. A longitudinal cohort study suggests that abnormal dietary behavior is significantly associated with the onset of AIS (OR = 3.23) (52), further supporting the possibility that picky eating habits serve as a risk factor for scoliosis. This association was identified among a large number of candidate variables assessed against 59 screening-positive cases, and it should therefore be regarded as hypothesis-generating despite its persistence under mutual adjustment. Because picky eating is a lifestyle habit that can be addressed at the family level, improving children’s dietary patterns merits evaluation within comprehensive scoliosis prevention strategies, but the causal nature of the association requires confirmation in prospective longitudinal studies.

Thumb-inward grip pen-holding posture (Type 3) was associated with suspected scoliosis within the domain of daily habits (OR = 2.137, 95% CI 1.076–4.243), but this association was attenuated to non-significance in the joint model (OR = 1.55, 95% CI 0.77–3.11, *p* = 0.221). To date no previous research has addressed this issue, and the mechanism outlined below is therefore presented as a hypothesis rather than as an established pathway. The biomechanical characteristics of the thumb-inward grip include excessive flexion and inward curling of the thumb, which forces the wrist joint into compensatory ulnar deviation, leading to increased forearm pronation and shoulder adduction, and ultimately an asymmetrical force distribution throughout the upper limb on the writing side. Under the daily cumulative writing load of several hours experienced by elementary school students, this persistent asymmetrical upper limb posture can transmit unbalanced tension from the shoulder and neck muscle groups to the cervical and thoracic spine, imposing asymmetrical rotational loads on the developing paraspinal muscles. Type 3 grip may also act as a marker of a child’s overall postural control rather than as a specific determinant. The development of a thumb-inward grip is often associated with insufficient postural awareness during the early establishment of writing habits, which may reflect weaker neuromuscular self-regulation in maintaining proper spinal alignment during daily learning activities (53). Two considerations argue for caution. First, 10 grip categories were examined against only 59 screening-positive cases, so this comparison is vulnerable to multiple testing and to sparse-data instability. Second, the attenuation was already present in an ordinary logistic model that ignored clustering, suggesting that Type 3 grip may partly serve as a marker of co-occurring risk factors (lower physical activity, picky eating and coexisting postural abnormalities) rather than as an independent determinant. The proposed biomechanical pathway was not examined in this study. This finding should therefore be regarded as hypothesis-generating and requires confirmation through direct observation of a standardized writing task and longitudinal follow-up before correction of pen-holding posture can be recommended as a preventive measure.

Regarding the learning environment, regular adjustment of chair and desk height (OR = 0.157) and regular rotation of classroom seating between left and right sides (OR = 0.204) were both associated with lower odds of suspected scoliosis, and both associations persisted in the joint model after adjustment for grade, postural indicators and classroom-level clustering (OR = 0.18 and OR = 0.19, respectively; Table 8), representing key findings of this study regarding the school learning environment. Numerous studies have confirmed that prolonged poor posture resulting from mismatched desk and chair heights relative to a child’s height is a risk factor associated with scoliosis (22). As children grow rapidly in height, regularly adjusting chair and desk height may reduce the asymmetric load on the spine associated with asymmetrical sitting postures. The protective mechanism of regularly rotating classroom seating may lie in disrupting factors—such as asymmetrical lighting and fixed viewing angles toward the blackboard—that induce habitual rotational misalignment of the cervical and upper thoracic spine in fixed seating arrangements. This may help balance the load on both sides of the spine and thereby reduce rotational stress on a single side. Currently, biomechanical evidence regarding these seat-related protective mechanisms remains insufficient and requires further research and validation. Both measures are low-cost and readily scalable in school settings and merit evaluation for inclusion in school-based scoliosis prevention policies. It should also be noted that both practices were reported by guardians for their own child rather than verified as school or class policy, so these variables may partly reflect the degree of parental attention to the child’s study environment in addition to the practices themselves.

### 4.5 Strengths and limitations

This study has the following strengths: (1) It employed a sound sampling method, resulting in a large sample size and comprehensive coverage across grade levels; the resulting epidemiological data can serve as a reference for the prevention and control of scoliosis in elementary schools in Northeast China; (2) Through pre-screening calibration and verification of inter-device agreement, a large-scale comparison between the E-scoliometer and M-scoliometer groups was conducted in a real-world school setting, providing evidence on the comparability of the positive screening rates and on the efficiency of the two screening tools; (3) Postural assessment and lifestyle questionnaires were incorporated into correlation analyses to explore risk factors associated with scoliosis; (4) The implementation of standardized training, inter-rater reliability assessment of pre-screening procedures, and multi-stage quality control throughout the study helped ensure the reliability of the measurement data.

This study has the following limitations: (1) The cross-sectional design cannot establish causality; all conclusions regarding risk factors are based on correlational evidence, and causal inferences depend on subsequent longitudinal studies; (2) Cases were defined by ATR-based screening positivity (“suspected scoliosis”) rather than radiographic confirmation (Cobb angle ≥ 10°), which is impractical for an entire screened population; the reported positive rate therefore reflects screening positivity rather than true prevalence, and the findings pertain to suspected rather than confirmed scoliosis. In addition, screening was conducted over a standardized single-layer garment rather than on the exposed back, reflecting the operating conditions of a large-scale program carried out on school premises rather than those of a clinical examination. The garment layer may have obscured subtle surface asymmetry and slightly altered device contact, and measurements close to the 5° threshold may therefore have been misclassified in either direction. Although the same garment conditions applied to both device groups, residual measurement error affecting the reported positive screening rates cannot be excluded. Future studies should incorporate radiographic or radiation-free ultrasound confirmation; (3) The comparison between the two devices was a test of difference rather than a formal equivalence trial: no equivalence margin was pre-specified and no *a priori* power calculation was performed. The comparable positive screening rates therefore indicate that no difference was detected rather than that equivalence has been established, and a formal equivalence study with a pre-specified margin is warranted. In the absence of a radiographic reference standard, the diagnostic accuracy of either device was not assessed. The main cohort comparison contrasted population-level screening yields between two independently allocated groups of classes, whereas individual-level agreement between the devices was demonstrated only in the pilot study. The comparison of screening efficiency is subject to two further constraints: the two groups were screened by different examiners drawn from the same trained personnel pool, so an examiner contribution to the observed difference cannot be separated from the device effect; and the interval was timed by stopwatch in the manual group and by the internal timer in the electronic group, although the resulting measurement error is small relative to the difference observed; (4) Postural assessment was not performed under blinded conditions. It preceded the Adams forward bend test and ATR measurement, so screening status was unknown at that point, but the same examiners conducted both procedures, and shoulder height asymmetry and flat back overlap with the surface signs evaluated during the general visual inspection. Differential ascertainment of these two indicators therefore cannot be excluded, their odds ratios may be inflated, and the apparent discrimination of the joint model is correspondingly optimistic. Assessment by examiners independent of the screening team is warranted in future studies; (5) Lifestyle data were derived from parent-reported questionnaires, which may carry risks of recall bias and information bias; pen-holding posture was classified with reference to a demonstration by the child and an illustrated card, but as this took place outside a standardized writing task, some misclassification of grip type remains possible; (6) Regular adjustment of chair and desk height and regular rotation of classroom seating were reported by guardians for each individual child rather than verified as school policy, and may therefore be subject to misclassification; (7) The risk factor analysis was based solely on a subsample from the manual screening group, and the representativeness of the conclusions is limited by the scope of the subsample; (8) With only 59 screening-positive cases, statistical power was limited and several confidence intervals remain wide. The joint model was restricted to nine parameters (6.56 events per variable), but its variables were selected on the basis of significance in the domain-specific models; the estimates are therefore conditional on that selection, were validated internally only, and quantify associations rather than predicting individual risk; (9) Clustering was addressed at the class level, with a school-level specification as a sensitivity analysis. Because only 10 schools were sampled, residual school-level confounding cannot be excluded; (10) Study participants were recruited from urban and suburban areas of Harbin; therefore, caution is warranted when generalizing these findings to other regions with significantly different climatic conditions, lifestyles, and socioeconomic backgrounds.

## 5 Conclusion

This study screened 11,587 elementary school students in grades 1 to 5 in Harbin. The overall positive screening rate for suspected scoliosis was 1.61%, with a significant increasing trend observed across grade levels. With regard to screening tool comparison, the positive screening rates and severity distributions of the E-scoliometer and the M-scoliometer were comparable, with no difference detected, while the E-scoliometer reduced measurement time by approximately 54%. In a joint model with mutual adjustment across domains and adjustment for classroom-level clustering, flat back, picky eating, insufficient daily physical exercise, mild wasting, shoulder height asymmetry and higher grade were associated with increased odds of suspected scoliosis, whereas regular adjustment of chair and desk height and regular rotation of classroom seating between left and right sides were associated with reduced odds. The association initially observed for thumb-inward grip pen-holding posture did not persist under mutual adjustment and is reported as hypothesis-generating. Because the design is cross-sectional and based on 59 screening-positive cases, these associations are correlational; nonetheless most of the factors identified are modifiable, and it is therefore recommended that electronic screening tools be integrated into the existing school-based screening system, and that ensuring adequate daily physical activity, improving children’s nutritional behaviors and standardizing classroom furniture and seating management be evaluated within a comprehensive scoliosis prevention and control strategy. Future research should further verify the causal nature of these associations through prospective longitudinal designs.

## Data Availability

The raw data supporting the conclusions of this article will be made available by the authors, without undue reservation.

## Glossary

AIS: Adolescent Idiopathic Scoliosis
APT: Anterior Pelvic Tilt
ATR: Angle of Trunk Rotation
BMI: Body Mass Index
CI: Confidence Interval
E-scoliometer: Electronic Scoliometer
FB: Flat Back
FHP: Forward Head Posture
ICC: Intraclass Correlation Coefficient
IGF-1: Insulin-like Growth Factor-1
IS: Idiopathic Scoliosis
LA: Leg Alignment
M-scoliometer: Manual Scoliometer
OR: Odds Ratio
SD: Standard Deviation
SHA: Shoulder Height Asymmetry
SSS: School-Based Scoliosis Screening
WHO: World Health Organization
WS: Winged Scapula
AUC: Area under the Receiver Operating Characteristic Curve
EPV: Events per Variable
GEE: Generalized Estimating Equation.
