# Supplementary material for: Application of the electronic scoliometer in school-based scoliosis screening among elementary school students and analysis of factors associated with suspected scoliosis
Source: Front Public Health. 2026 Sep 21;14:1926931. doi: 10.3389/fpubh.2026.1926931 (PMC13635355; doi:10.3389/fpubh.2026.1926931)
Supplement: Supplementary file 1 [file Supplementary_file_1.DOCX]

Supplementary Materials

[**Figure S1.** Study design and participant flow.	2](#_Toc236926507)

[**Figure S2.** Schematic illustration of the four-step standardized scoliosis screening procedure.	3](#_Toc236926508)

[**Figure S3.** Illustrated reference card used to classify pen-holding posture into ten grip types.	4](#_Toc236926509)

[**Table S1.** Five-Point Cooperation Scoring Rubric for School-Based Scoliosis Screening	5](#_Toc236926510)

[**Table S2.** Sex- and Age-Specific BMI Cut-Off Values for Nutritional Status Classification Among School-Age Children	6](#_Toc236926511)

[**Table S3.** Assessment Criteria for Seven Postural Indicators Evaluated During School-Based Scoliosis Screening	8](#_Toc236926512)

[**Table S4.** Questionnaires Used in School-Based Scoliosis Screening Study	12](#_Toc236926513)

[**Table S5.** Sensitivity analyses of the joint model of factors associated with suspected scoliosis	14](#_Toc236926514)

[**Table S6.** Number of parameters estimated and events per variable for each multivariable model	15](#_Toc236926515)

**Table S7.** Participant flow and completeness of the analytic samples ………………………..16

**Figure S1.** Study design and participant flow.

Note. Eligibility and guardian consent were determined before class allocation and are therefore shown for the cohort as a whole. Pupils excluded after examination were those whose cooperation score of 1 precluded a valid ATR measurement. The risk-factor subsample was drawn from the M-scoliometer group; the exclusions shown did not overlap. Detailed participant flow is reported in Supplementary Materials Table S7. ATR = angle of trunk rotation; M-scoliometer = manual scoliometer; E-scoliometer = electronic scoliometer.

**Figure S2.** Schematic illustration of the four-step standardized scoliosis screening procedure.

Note. Step 4 (Spinal Movement Test) was performed when the general visual inspection was abnormal, the Adams forward bend test was positive, or the maximum ATR was ≥ 5°. Each movement was performed twice (×2), after which the general visual inspection and the ATR measurement were repeated; the values obtained on this second measurement determined the final screening classification. ATR = angle of trunk rotation.

**Figure S3.** Illustrated reference card used to classify pen-holding posture into ten grip types.

Note. This card was displayed and explained in person to each guardian at the time of questionnaire completion. Type numbering and descriptions correspond to item Q3 in Supplementary Materials Table S4.

**Table S1.** Five-Point Cooperation Scoring Rubric for School-Based Scoliosis Screening

| **Score** | **Level & Definition** | **Behavioral Criteria** | **Operational Notes** |
| --- | --- | --- | --- |
| **5** | **EXCELLENT**  Fully compliant; no verbal reminder or physical guidance required. | Assumed correct Adams position on first instruction.  Remained completely motionless during ATR measurement at all three spinal levels.  Responded immediately to all examiner instructions without repetition.  No signs of distress, anxiety, or behavioral resistance. | Examination completed without interruption or repositioning. |
| **4** | **GOOD**  Largely compliant; required one verbal reminder only; no physical guidance. | Correct position achieved after one brief verbal prompt.  Minor, self-corrected positional deviation (e.g., knee flexion ≤5°).  Calm and cooperative; minimal initial hesitancy resolved without intervention. | No measurement step repeated; no additional time beyond protocol. |
| **3** | **MODERATE**  Partially compliant; 2–3 verbal reminders required; no physical guidance; minor delay. | Required 2–3 verbal reminders to achieve or maintain correct forward bend posture.  Intermittent fidgeting necessitated repetition of ≥1 ATR measurement.  Mild anxiety or restlessness, manageable without physical intervention. | Examination completed with delay ≤30 s beyond standard protocol duration. |
| **2** | **POOR**  Partially compliant; physical guidance required in addition to ≥4 verbal reminders; notable delay. | Physical guidance required (e.g., manual knee extension assistance) alongside ≥4 verbal reminders.  Significant movement or resistance; ≥2 measurement steps repeated.  Marked distress (e.g., crying, verbal refusal) substantially impeding examination. | Examination completed with delay >30 s or required physical intervention; data usability at examiner's discretion. |
| **1** | **NON-COOPERATIVE**  Unable to complete examination; valid ATR measurement could not be obtained despite maximal effort. | Unable to maintain any approximation of the required Adams forward bend position.  Extreme resistance (e.g., sustained crying, active withdrawal) precluded valid ATR measurement.  Examination terminated or all data deemed invalid. | No valid ATR recorded. Case documented as non-evaluable and excluded per pre-specified protocol. |

Note. A single cooperation score (1–5) was assigned by the examining clinician to each participant immediately after completion of the Adams forward bend test and ATR measurement, based on direct behavioral observation throughout the screening session. Participants assigned a score of 1 (non-cooperative) were excluded from all screening outcome analyses per pre-specified protocol criteria. ATR = angle of trunk rotation.

**Table S2.** Sex- and Age-Specific BMI Cut-Off Values for Nutritional Status Classification Among School-Age Children

| **Part A. Boys** | | | | |
| --- | --- | --- | --- | --- |
| **Age (years)** | **Overweight** | **Obesity** | **Mild wasting** | **Moderate to severe wasting** |
|  | *(BMI, kg/m²)* | *(BMI, kg/m²)* | *(BMI range, kg/m²)* | *(BMI cut-off, kg/m²)* |
| 6.0– | 16.4 | 17.7 | 13.3–13.4 | ≤13.2 |
| 6.5– | 16.7 | 18.1 | 13.5–13.8 | ≤13.4 |
| 7.0– | 17.0 | 18.7 | 13.6–13.9 | ≤13.5 |
| 7.5– | 17.4 | 19.2 | 13.6–13.9 | ≤13.5 |
| 8.0– | 17.8 | 19.7 | 13.7–14.0 | ≤13.6 |
| 8.5– | 18.1 | 20.3 | 13.7–14.0 | ≤13.6 |
| 9.0– | 18.5 | 20.8 | 13.8–14.1 | ≤13.7 |
| 9.5– | 18.9 | 21.4 | 13.9–14.2 | ≤13.8 |
| 10.0– | 19.2 | 21.9 | 14.0–14.4 | ≤13.9 |
| 10.5– | 19.6 | 22.5 | 14.1–14.6 | ≤14.0 |
| 11.0– | 19.9 | 23.0 | 14.3–14.9 | ≤14.2 |
| 11.5– | 20.3 | 23.6 | 14.4–15.1 | ≤14.3 |
| 12.0– | 20.7 | 24.1 | 14.5–15.4 | ≤14.4 |
| 12.5– | 21.0 | 24.7 | 14.6–15.6 | ≤14.5 |
| 13.0– | 21.4 | 25.2 | 14.9–15.9 | ≤14.8 |
| 13.5– | 21.9 | 25.7 | 15.1–16.1 | ≤15.0 |
| 14.0– | 22.3 | 26.1 | 15.4–16.4 | ≤15.3 |
| 14.5– | 22.6 | 26.4 | 15.6–16.7 | ≤15.5 |
| 15.0– | 22.9 | 26.6 | 15.9–16.9 | ≤15.8 |
| 15.5– | 23.1 | 26.9 | 16.1–17.0 | ≤16.0 |
| 16.0– | 23.3 | 27.1 | 16.3–17.3 | ≤16.2 |
| 16.5– | 23.5 | 27.4 | 16.5–17.5 | ≤16.4 |
| 17.0– | 23.7 | 27.6 | 16.7–17.7 | ≤16.6 |
| 17.5– | 23.8 | 27.8 | 16.9–17.9 | ≤16.8 |
| 18.0 | 24.0 | 28.0 | — | — |
| **Part B. Girls** | | | | |
| **Age (years)** | **Overweight** | **Obesity** | **Mild wasting** | **Moderate to severe wasting** |
|  | *(BMI, kg/m²)* | *(BMI, kg/m²)* | *(BMI range, kg/m²)* | *(BMI cut-off, kg/m²)* |
| 6.0– | 16.2 | 17.5 | 12.9–13.1 | ≤12.8 |
| 6.5– | 16.5 | 18.0 | 13.0–13.3 | ≤12.9 |
| 7.0– | 16.8 | 18.5 | 13.1–13.4 | ≤13.0 |
| 7.5– | 17.2 | 19.0 | 13.1–13.5 | ≤13.0 |
| 8.0– | 17.6 | 19.4 | 13.2–13.6 | ≤13.1 |
| 8.5– | 18.1 | 19.9 | 13.2–13.7 | ≤13.1 |
| 9.0– | 18.5 | 20.4 | 13.3–13.8 | ≤13.2 |
| 9.5– | 19.0 | 21.0 | 13.3–13.9 | ≤13.2 |
| 10.0– | 19.5 | 21.5 | 13.4–14.0 | ≤13.3 |
| 10.5– | 20.0 | 22.1 | 13.5–14.1 | ≤13.4 |
| 11.0– | 20.5 | 22.7 | 13.8–14.3 | ≤13.7 |
| 11.5– | 21.1 | 23.3 | 14.0–14.5 | ≤13.9 |
| 12.0– | 21.5 | 23.9 | 14.2–14.7 | ≤14.1 |
| 12.5– | 21.9 | 24.5 | 14.4–14.9 | ≤14.3 |
| 13.0– | 22.2 | 25.0 | 14.7–15.3 | ≤14.6 |
| 13.5– | 22.6 | 25.6 | 15.0–15.6 | ≤14.9 |
| 14.0– | 22.8 | 25.9 | 15.4–16.0 | ≤15.3 |
| 14.5– | 23.0 | 26.3 | 15.8–16.3 | ≤15.7 |
| 15.0– | 23.2 | 26.6 | 16.1–16.6 | ≤16.0 |
| 15.5– | 23.4 | 26.9 | 16.3–16.8 | ≤16.2 |
| 16.0– | 23.6 | 27.1 | 16.5–17.0 | ≤16.4 |
| 16.5– | 23.7 | 27.4 | 16.6–17.1 | ≤16.5 |
| 17.0– | 23.8 | 27.6 | 16.7–17.2 | ≤16.6 |
| 17.5– | 23.9 | 27.8 | 16.8–17.3 | ≤16.7 |
| 18.0 | 24.0 | 28.0 | — | — |

Note. BMI = body mass index (kg/m²). Overweight and obesity cut-offs are sex- and age-specific values derived from the Chinese national standard WS/T 586–2018 (Screening for Overweight and Obesity Among School-Age Children and Adolescents). Wasting cut-offs are derived from WS/T 456–2014 (Screening for Malnutrition Among School-Age Children and Adolescents). Wasting reference values at age 18.0 years are not specified in WS/T 456–2014 (denoted as —). Category definitions (exact inequalities): Moderate to severe wasting, BMI ≤ lower wasting cut-off; Mild wasting, lower wasting cut-off < BMI ≤ upper wasting cut-off; Normal weight, upper wasting cut-off < BMI < overweight cut-off; Overweight, overweight cut-off ≤ BMI < obesity cut-off; Obesity, BMI ≥ obesity cut-off.

**Table S3.** Assessment Criteria for Seven Postural Indicators Evaluated During School-Based Scoliosis Screening

| **1. Forward Head Posture (FHP)** | |
| --- | --- |
| **Definition** | Anterior displacement of the head relative to the trunk in the sagittal plane, with the external auditory meatus positioned visibly anterior to the acromion. |
| **Examination Method** | The participant stands in a relaxed natural posture. The examiner visually assesses the sagittal profile from the lateral aspect, comparing the position of the tragus to that of the shoulder (acromion). No instruments are required. Confirmation may be obtained by asking the participant to stand with heels, buttocks, and shoulder blades against a flat wall. |
| **Positive Criterion (Abnormal)** | The tragus is visibly anterior to the acromion on lateral inspection; or, when standing against the wall (heels, buttocks, and shoulder blades contacting the wall), the back of the head fails to contact the wall. |
| **Clinical Relevance** | FHP is prevalent in school-age children and is associated with increased cervical spinal load and compensatory thoracic hyperkyphosis. In scoliosis screening, FHP may co-occur with or amplify other postural abnormalities relevant to spinal assessment. |
| **Reference(s)** | [S1, S2] |
| **2. Shoulder Height Asymmetry (SHA)** | |
| **Definition** | Visible inequality in the bilateral shoulder girdle heights in the coronal plane, with one shoulder clearly higher or lower than the other. |
| **Examination Method** | The participant stands with their back to the examiner, feet together, arms relaxed at sides. The examiner visually compares the height of bilateral shoulders (at the level of the superior trapezius / acromion) from the posterior view. |
| **Positive Criterion (Abnormal)** | Visible height difference between bilateral shoulder tops on posterior inspection, with one shoulder clearly higher or lower than the other. |
| **Clinical Relevance** | Shoulder height asymmetry is one of the cardinal clinical signs of adolescent idiopathic scoliosis (AIS) and is frequently the first sign identified during school screening. It is a standard component of the Scoliosis Research Society (SRS) examination protocol. |
| **Reference(s)** | [S3, S4] |
| **3. Winged Scapula (WS)** | |
| **Definition** | Abnormal posterior protrusion of the medial border or inferior angle of one or both scapulae away from the thorax, producing a 'wing-like' appearance on posterior inspection. |
| **Examination Method** | The participant stands with their back to the examiner, arms relaxed at sides. The examiner inspects from the posterior view and observes whether the medial border or inferior angle of either scapula protrudes noticeably away from the thoracic wall. No instruments are required. |
| **Positive Criterion (Abnormal)** | Medial border or inferior angle of one or both scapulae visibly protrudes posteriorly away from the thorax on posterior inspection. |
| **Clinical Relevance** | Winged scapula may indicate serratus anterior weakness or altered scapulothoracic mechanics. In the context of scoliosis screening, it may serve as a surface sign of asymmetric vertebral rotation or coronal imbalance. |
| **Reference(s)** | [S4, S5] |
| **4. Kyphosis** | |
| **Definition** | Excessive forward curvature of the thoracic spine resulting in a visibly rounded upper back ('humpback' appearance) when viewed from the lateral aspect. The normal thoracic kyphosis range is 20–45°; clinically significant kyphosis exceeds this range. |
| **Examination Method** | The participant stands in a relaxed natural posture. The examiner observes the sagittal profile from the lateral view, assessing the degree of upper back rounding. The participant is then asked to stand as straight as possible: postural kyphosis corrects voluntarily; structural kyphosis (e.g., Scheuermann's disease) does not fully correct. |
| **Positive Criterion (Abnormal)** | Visibly excessive rounding of the upper back on lateral inspection, with or without full voluntary correction when the participant is asked to stand straight. |
| **Clinical Relevance** | Thoracic kyphosis commonly co-occurs with scoliosis and may reflect Scheuermann's disease or habitual poor posture. Assessment of sagittal plane alignment complements the coronal plane evaluation during scoliosis screening. |
| **Reference(s)** | [S6, S7] |
| **5. Flat Back (FB)** | |
| **Definition** | Absence or marked reduction of the normal lumbar lordosis (inward curvature of the lower back), causing the lumbar region to appear abnormally straight or flattened on lateral inspection. |
| **Examination Method** | The participant stands in a relaxed natural posture. The examiner observes the sagittal profile from the lateral view, assessing whether the normal inward curve of the lumbar spine is present. A practical supplementary check: the participant stands with heels and shoulder blades against a wall; a reduced or absent space between the lower back and the wall indicates flat back. |
| **Positive Criterion (Abnormal)** | The lumbar region appears visibly straight or flattened on lateral inspection, with the normal inward curvature markedly reduced or absent. |
| **Clinical Relevance** | Flat back (reduced lumbar lordosis) is associated with posterior pelvic tilt and altered spinal sagittal balance. Assessment of lumbar sagittal contour provides important complementary information during school-based scoliosis screening. |
| **Reference(s)** | [S8] |
| **6. Anterior Pelvic Tilt (APT)** | |
| **Definition** | Forward rotation of the pelvis, characterized by the anterior superior iliac spine (ASIS) tilting downward relative to the posterior superior iliac spine (PSIS), accompanied by exaggerated lumbar lordosis, forward protrusion of the abdomen, and posterior protrusion of the buttocks. |
| **Examination Method** | The participant stands in a relaxed natural posture. The examiner observes the lateral profile and assesses three visual signs: (1) exaggerated lumbar lordosis (excessive lower back arch); (2) forward protrusion of the lower abdomen; and (3) posterior protrusion of the buttocks. All three features are assessed by direct visual inspection. |
| **Positive Criterion (Abnormal)** | Visibly exaggerated lumbar lordosis with an obvious forward tilt of the pelvis—manifested as prominent posterior buttock protrusion and forward abdominal protrusion—on lateral inspection. |
| **Clinical Relevance** | A large-scale case-control study of 2,538 participants found that standing with anterior pelvic tilt was independently associated with a 173% increased risk of AIS (OR = 2.73, 95% CI: 1.41–5.28), making it one of the strongest postural risk factors for scoliosis identified in the current literature. |
| **Reference(s)** | [S9] |
| **7. Leg Alignment (LA) (Normal / X-type / O-type / XO-type)** | |
| **Definition** | Coronal plane alignment of the lower extremities classified into four categories: Normal (straight alignment); X-type (genu valgum / knock-knee: knees in contact but ankles apart); O-type (genu varum / bowleg: ankles in contact but knees apart); XO-type (mixed valgus/varus pattern within the same or across limbs). |
| **Examination Method** | The participant stands with feet together (medial malleoli approximated) and patellae facing forward. The examiner inspects from the anterior view and classifies the knee-ankle relationship into one of the four categories. Recorded as a 4-category variable (not a binary outcome). |
| **Positive Criterion (Abnormal)** | Any of X-type, O-type, or XO-type. In children aged ≥7 years (this study population: 6–12 years), physiologic alignment should have normalized. Persistent genu valgum (intermalleolar distance >8 cm) or genu varum (intercondylar distance >5 cm) in the standing position after age 7 is considered pathological. |
| **Clinical Relevance** | Lower extremity alignment directly influences pelvic tilt and lumbar spinal loading. Persistent genu varum or valgum beyond the physiologic window may create leg length discrepancy and secondary functional scoliosis, providing a rationale for inclusion in the postural assessment battery. |
| **Reference(s)** | [S10, S11] |

*Note.* All seven indicators were assessed by visual inspection during standing evaluation by trained examiners, prior to the Adams forward bend test and ATR measurement. No instruments were required; all assessments were conducted in real time during mass school screening. Indicators 1–6 (FHP, SHA, WS, kyphosis, FB, and APT) were recorded as binary outcomes (normal/abnormal); indicator 7 (LA) was recorded as a four-category variable (normal, X-type, O-type, and XO-type). FHP = forward head posture; SHA = shoulder height asymmetry; WS = winged scapula; FB = flat back; APT = anterior pelvic tilt; LA = leg alignment; AIS = adolescent idiopathic scoliosis; SRS = Scoliosis Research Society; ATR = angle of trunk rotation; ASIS = anterior superior iliac spine; PSIS = posterior superior iliac spine; OR = odds ratio; CI = confidence interval.

**References**

[S1] Raine S, Twomey LT. Head and shoulder posture variations in 160 asymptomatic women and men. *Arch Phys Med Rehabil.* 1997;78(11):1215–1223.

[S2] Hansraj KK. Assessment of stresses in the cervical spine caused by posture and position of the head. *Surg Technol Int.* 2014;25:277–279.

[S3] Watson AW, MacDonncha C. A reliable technique for the assessment of posture: assessment criteria for aspects of posture. J Sports Med Phys Fitness. 2000;40(3):260–270.

[S4] Negrini S, Donzelli S, Aulisa AG, et al. 2016 SOSORT guidelines: orthopaedic and rehabilitation treatment of idiopathic scoliosis during growth. Scoliosis Spinal Disord. 2018;13:3.

[S5] Struyf F, Nijs J, Mottram S, et al. Clinical assessment of the scapula: a review of the literature. *Br J Sports Med.* 2014;48(11):883–890.

[S6] Voutsinas SA, MacEwen GD. Sagittal profiles of the spine. Clin Orthop Relat Res. 1986;(210):235–242.

[S7] Sardar ZM, Ames RJ, Lenke L. Scheuermann's kyphosis: diagnosis, management, and selecting fusion levels. J Am Acad Orthop Surg. 2019;27(10):e462–e472. doi:10.5435/JAAOS-D-17-00748.

[S8] Bernhardt M, Bridwell KH. Segmental analysis of the sagittal plane alignment of the normal thoracic and lumbar spines and thoracolumbar junction. *Spine (Phila Pa 1976).* 1989;14(7):717–721.

[S9] Herrington L. Assessment of the degree of pelvic tilt within a normal asymptomatic population. Man Ther. 2011;16(6):646–648.

[S10] Cheng JCY, Chan PS, Chiang SC, Hui PW. Angular and rotational profile of the lower limb in 2,630 Chinese children. *J Pediatr Orthop.* 1991;11(2):154–161.

[S11] Baird DC, Dickison CG, Spires HI. Lower Extremity Abnormalities in Children. Am Fam Physician. 2025;111(2):125-139.

**Table S4.** Questionnaires Used in School-Based Scoliosis Screening Study

| **School:** ________________________ **Grade:** ______ **Class:** ______ **Date:** ____/____/______  **Child's name:** ________________________________ | |
| --- | --- |
| **Dear Parent / Legal Guardian** | |
| Thank you for participating in this scoliosis screening study. This short questionnaire asks about your child's daily habits and school environment. It takes approximately 5 minutes to complete. All information is anonymous and used solely for research purposes. Please answer all questions based on your child's actual situation. A research team member is available to assist if needed.  **Legal guardian signature:** ______________________________ **Relationship to child:** _____________________ | |
| **Section A: Daily Habits** | |
| **Q1. On a typical school day, how many hours does your child spend using electronic devices?** | |
| □ | Less than 1 hour per day |
| □ | 1 to 2 hours per day |
| □ | More than 2 hours per day |
| **Q2. On a typical school day, how many hours does your child spend on physical exercise or active outdoor play?** | |
| □ | Less than 1 hour per day |
| □ | 1 to 2 hours per day |
| □ | More than 2 hours per day |
| **Q3. How does your child usually hold a pen or pencil when writing? Please select the type that best matches, referring to the illustrated guide shown by the research staff.** | |
| □ | Type 0 — Correct grip: pen held between thumb and index finger, supported by middle finger underneath; wrist straight; pen angled about 45–60° to the paper |
| □ | Type 1 — Thumb-over grip: thumb overlaps and covers the index finger from above |
| □ | Type 2 — Full-fist grip: all fingers wrap around the pen like a fist |
| □ | Type 3 — Thumb-inward grip: thumb is excessively bent inward toward the palm |
| □ | Type 4 — Index-push grip: index finger pushes against the pen with excessive sideways pressure |
| □ | Type 5 — Index-curl grip: index finger is markedly curled underneath the pen |
| □ | Type 6 — Grip too close to tip: fingers are less than 1 cm from the pen nib |
| □ | Type 7 — Grip too far from tip: fingers are more than 4 cm from the pen nib |
| □ | Type 8 — Wrist-deviated grip: wrist is noticeably bent sideways while writing |
| □ | Type 9 — Other unusual grip (please describe if possible): _______________________________ |
| **Q4. Has your child had an eye examination? If yes, is there a significant difference in the degree of short-sightedness (myopia) between the left and right eyes?** | |
| □ | No significant difference between the two eyes, or no short-sightedness in either eye |
| □ | Yes, there is a notable difference of 100 degrees (1.00 diopter) or more between the two eyes |
| **Q5. Does your child have picky eating habits — for example, persistently refusing to eat vegetables, meat, dairy, eggs, or other major food groups?** | |
| □ | No — the child eats a reasonably varied diet |
| □ | Yes — the child regularly refuses one or more major food groups |
| **Q6. How would you describe your child's usual posture when standing freely?** | |
| □ | Always stands with good posture — weight evenly on both feet, upright |
| □ | Occasionally shifts or adjusts stance |
| □ | Stands however feels comfortable — no attention to posture |
| **Q7. What is the firmness of the mattress your child mainly sleeps on?** | |
| □ | Medium — neither too hard nor too soft |
| □ | Soft — the body sinks noticeably into the mattress |
| □ | Firm — very hard surface |
| **Q8. Does your child attend any extracurricular classes or activities outside school hours?** | |
| □ | None |
| □ | Academic or arts classes only (e.g., tutoring, music, art) |
| □ | Sports or physical activity classes only (e.g., swimming, gymnastics, martial arts, dance) |
| □ | Both academic and sports classes |
| **Section B: School Environment** | |
| **Q9. To the best of your knowledge, does your child's school regularly adjust the height of the chair and desk as the child grows?** | |
| □ | Yes — the school adjusts furniture height regularly |
| □ | No — the furniture height is rarely or never changed |
| **Q10. To the best of your knowledge, does your child's teacher regularly rotate seating positions between the left and right sides of the classroom?** | |
| □ | Yes — seating is regularly rotated between the left and right sides |
| □ | No — the child always sits on the same side of the classroom |
| **Thank you for your time and cooperation.** | |

Note. This questionnaire was administered to legal guardians immediately following posture assessment on the day of screening. A trained research team member explained all items verbally to each guardian prior to completion to ensure consistent understanding. All questionnaires were reviewed item-by-item upon collection; missing or logically inconsistent responses were clarified and corrected on the spot, and all forms were double-checked before database entry. For Q3 (pen-holding posture), the child demonstrated their usual writing grip in the presence of the guardian, who then identified the matching type from a standardized illustrated reference card depicting all 10 grip types (Figure S3); the card was displayed and explained in person by a trained research team member.

**Table S5.** Sensitivity analyses of the joint model of factors associated with suspected scoliosis

| **Variable** | **GEE, exchangeable,clustered by class(primary analysis)OR, P** | **GEE, independent,cluster-robust SEOR, P** | **GEE, exchangeable,clustered by schoolOR, P** |
| --- | --- | --- | --- |
| Grade (per one-grade increment) | 1.43, <0.001 | 1.41, <0.001 | 1.41, <0.001 |
| Mild wasting | 3.80, 0.004 | 3.87, 0.003 | 3.67, 0.027 |
| Shoulder height asymmetry | 3.06, <0.001 | 3.00, <0.001 | 3.10, <0.001 |
| Flat back | 7.36, <0.001 | 7.39, <0.001 | 7.43, <0.001 |
| Daily physical exercise < 1 h/day | 3.86, <0.001 | 3.85, <0.001 | 3.78, <0.001 |
| Thumb-inward pen grip (Type 3) | 1.55, 0.221 | 1.49, 0.275 | 1.56, 0.173 |
| Picky eating | 4.19, <0.001 | 4.01, <0.001 | 4.09, <0.001 |
| Regular adjustment of chair and desk height | 0.18, <0.001 | 0.17, <0.001 | 0.19, <0.001 |
| Regular rotation of classroom seating | 0.19, <0.001 | 0.19, <0.001 | 0.20, <0.001 |

**Note.** OR = odds ratio; SE = standard error; GEE = generalized estimating equation. All three specifications contain the same nine variables, entered simultaneously as binary indicators, and all use robust (“sandwich”) variance estimation. The primary analysis (first column) is reported with full 95% confidence intervals in Table 8 of the main text. All seven robust factors retained P < 0.05 under every specification, and thumb-inward pen grip (Type 3) remained non-significant under all three (P = 0.173–0.371). P < 0.05 was considered statistically significant.

**Table S6.** Number of parameters estimated and events per variable for each multivariable model

| **Model** | **Domain** | **Parametersestimated** | **Events** | **EPV** | **Model performance** |
| --- | --- | --- | --- | --- | --- |
| Table 5 | Demographic characteristics and postural indicators | 10 | 59 | 5.9 | Not assessed; reported for within-domain description only |
| Table 7 | Daily habits and school learning environment | 10 | 59 | 5.9 | Not assessed; reported for within-domain description only |
| Table 8 | Both domains, mutually adjusted, class-clustered (primary analysis) | 9 | 59 | 6.56 | AUC 0.915; optimism-corrected AUC 0.910; Hosmer–Lemeshow χ² = 2.77, P = 0.948; Brier score 0.0129 (reference 0.0161) |

**Note.** EPV = events per variable, calculated as the number of screening-positive cases divided by the number of parameters estimated (excluding the intercept); AUC = area under the receiver operating characteristic curve. The models in Tables 5 and 7 were fitted separately within domains and are therefore not adjusted across domains. The model in Table 8 is the primary risk-factor analysis: it contains variables from both domains simultaneously and accounts for within-class correlation using generalized estimating equations with an exchangeable working correlation structure and robust variance estimation. Optimism was estimated from 1,000 bootstrap resamples. The reference Brier score is that of a non-informative model assigning the overall prevalence (1.64%) to every pupil.

**Table S7.** Participant flow and completeness of the analytic samples

**Panel A. Screening cohort**

| **Stage** | **M-scoliometer** | **E-scoliometer** | **Total** |
| --- | --- | --- | --- |
| Pupils enrolled in grades 1–5 at the 10 sampled schools | — | — | 11,668 |
| Ineligible: unable to complete the Adams forward bend test | — | — | 22 |
| Eligible | — | — | 11,646 |
| Guardian consent not obtained | — | — | 0 |
| Consented and allocated by class | 5,730 | 5,916 | 11,646 |
| Absent on the screening day | 28 | 18 | 46 |
| Examined | 5,702 | 5,898 | 11,600 |
| Excluded after examination: cooperation score 1, no valid ATR obtained | 5 | 8 | 13 |
| **Analyzed for the screening outcome** | **5,697** | **5,890** | **11,587** |

**Panel B. Risk-factor subsample**

| **Stage** | **n** |
| --- | --- |
| Classes sampled (2 per grade per school × 10 schools) | 100 |
| Pupils in sampled classes within the M-scoliometer analytic set | 3,674 |
| Postural assessment not completed | 3 |
| Questionnaire returned but unusable on verification | 71 |
| **Analyzed in the risk-factor subsample** | **3,600** |

**Note.** Eligibility and guardian consent were determined before class allocation and are therefore reported for the cohort as a whole. Stages are listed in the order in which they occurred; class allocation preceded the screening day. The exclusions listed in Panel B did not overlap, and no pupil was counted more than once. All variables were complete for the 3,600 pupils analysed; analyses were based on complete cases and no values were imputed. ATR = angle of trunk rotation.
